# Host lifestyle affects human microbiota on daily timescales

**DOI:** 10.1186/gb-2014-15-7-r89

**Published:** 2014-07-25

**Authors:** Lawrence A David, Arne C Materna, Jonathan Friedman, Maria I Campos-Baptista, Matthew C Blackburn, Allison Perrotta, Susan E Erdman, Eric J Alm

**Affiliations:** Society of Fellows, Harvard University, Cambridge, MA 02138 USA; FAS Center for Systems Biology, Harvard University, Cambridge, MA 02138 USA; QIAGEN Aarhus A/S, Silkeborgvej 2, 8000 Aarhus C, Denmark; Computational & Systems Biology, Massachusetts Institute of Technology, Cambridge, MA 02139 USA; Koch Institute for Integrative Cancer Research, Massachusetts Institute of Technology, Cambridge, MA 02139 USA; Institute of Bioengineering, School of Engineering, École Polytechnique Fédérale de Lausanne, CH-1015 Lausanne, Switzerland; Department of Civil & Environmental Engineering, Massachusetts Institute of Technology, Cambridge, MA 02139 USA; Division of Comparative Medicine, Massachusetts Institute of Technology, Cambridge, MA 02139 USA; Department of Biological Engineering, Massachusetts Institute of Technology, Cambridge, MA 02139 USA; The Broad Institute of MIT and Harvard, Cambridge, MA 02139 USA; Molecular Genetics & Microbiology and Center for Genomic & Computational Biology, Duke University, Durham, NC 27708 USA

## Abstract

**Background:**

Disturbance to human microbiota may underlie several pathologies. Yet, we lack a comprehensive understanding of how lifestyle affects the dynamics of human-associated microbial communities.

**Results:**

Here, we link over 10,000 longitudinal measurements of human wellness and action to the daily gut and salivary microbiota dynamics of two individuals over the course of one year. These time series show overall microbial communities to be stable for months. However, rare events in each subjects’ life rapidly and broadly impacted microbiota dynamics. Travel from the developed to the developing world in one subject led to a nearly two-fold increase in the Bacteroidetes to Firmicutes ratio, which reversed upon return. Enteric infection in the other subject resulted in the permanent decline of most gut bacterial taxa, which were replaced by genetically similar species. Still, even during periods of overall community stability, the dynamics of select microbial taxa could be associated with specific host behaviors. Most prominently, changes in host fiber intake positively correlated with next-day abundance changes among 15% of gut microbiota members.

**Conclusions:**

Our findings suggest that although human-associated microbial communities are generally stable, they can be quickly and profoundly altered by common human actions and experiences.

**Electronic supplementary material:**

The online version of this article (doi:10.1186/gb-2014-15-7-r89) contains supplementary material, which is available to authorized users.

## Background

The temporal dynamics of host-associated microbial communities (the microbiota) are of growing interest due to these communities’ relevance for health [1–5]. Normally, human microbiota remain stable for months, and possibly even years [6–8]. However, studies across mice and humans suggest that common aspects of the modern Western lifestyle, including antibiotics [1, 9–11] and high-fat diets [2], can persistently alter commensal microbial communities. In turn, those microbial disturbances may increase pathogen susceptibility [3], obesity [4, 12], and auto-inflammatory disease [5], maladies which are becoming more frequent in the developed world.

In spite of their potential health impact, a full list of lifestyle factors capable of altering human microbiota remains incomplete. Interventional studies are regularly performed to identify host behaviors that affect microbial dynamics, and they have notably demonstrated human gut microbial sensitivity to antibiotics [9–11], bowel surgery [13], and short-term diet shifts [14, 15]. However, interventions by design only test a small number of hypotheses; thus, a large, and potentially unfeasible, number of interventional studies are needed to fully explore the rich diversity of human actions and behaviors.

An alternative approach for efficiently linking numerous host factors to microbial responses is to longitudinally observe both the host and microbiota, and to infer relationships between them. Such observational studies have recently been used to show that menstrual cycles are the primary driver of vaginal microbial dynamics in women [16], and to show that infant gut microbiota begin transitioning towards adult communities after weaning [17]. In these time series, the quantity of host lifestyle variables that can be related to microbial dynamics is only bound by the number of host factors that can be tracked. Still, host tracking is non-trivial for ethical and logistical reasons, such as the need to repeatedly survey participants and the enforcement of subject compliance. Hence, many microbiome time series have featured limited longitudinal host metadata [8, 18], making it difficult to link microbial dynamics to host behavior.

Here, we address the dearth of coupled longitudinal datasets of human lifestyle and microbiota by tracking individuals and their commensal microbial communities each day over the course of 1 year. To let subjects comprehensively record their daily lives, we equipped them with iOS devices and a diary app that we configured to simplify personal record keeping. Paired with a simple diet record parsing algorithm that we wrote, this app allowed subjects to record data each day across 349 health and lifestyle variables spanning fitness, diet, exercise, bowel movements, mood, and illness (see Additional file 1 for a full list of measured variables). Even with our streamlined diary tools, we anticipated self-tracking to be inconvenient, and so we screened for study participants who would reliably collect daily records. Our screening yielded a small cohort of two healthy, unrelated male volunteers (Subjects A and B; see Additional file 2 for more demographic information). Yet, despite this small cohort size, the 10,124 measurements of subjects’ daily activity collected over the course of 1 year provides an unprecedented window into the health and lifestyle factors potentially regulating human-associated microbial environments.

**Figure 1:**
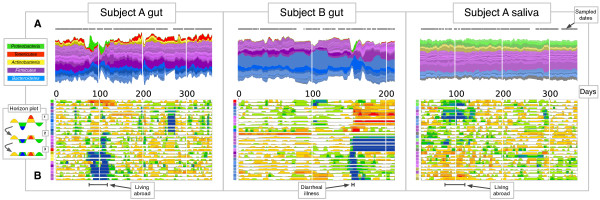
**Gut and salivary microbiota dynamics in two subjects over 1 year. (A)** Stream plots showing OTU fractional abundances over time. Each stream represents an OTU and streams are grouped by phylum: Firmicutes (purple), Bacteroidetes (blue), Proteobacteria (green), Actinobacteria (yellow), and Tenericutes (red). Stream widths reflect relative OTU abundances at a given time point. Sampled time points are indicated with gray dots over each stream plot. **(B)** Horizon graphs of most common OTUs’ abundance over time. Horizon graphs enable rapid visual comparisons between numerous time series [21]. Graphs are made by first median-centering each OTU time series and dividing the curve into colored bands whose width is the median absolute deviation (Inset, step 1). Next, the colored bands are overlaid (step 2) and negative values are mirrored upwards (step 3). Thus, warmer regions indicate date ranges where a taxon exceeds its median abundance, and cooler regions denote ranges where a taxon falls below its median abundance. Colored squares on the vertical axis correspond to stream colors in **(A)**. Time series in both the stream plots and horizon graphs were smoothed using Tukey’s running median. Lower black bars span Subject A’s travel abroad (days 71 to 122) and Subject B’s *Salmonella* infection (days 151 to 159).

Each day, subjects were asked to collect stool and saliva samples in order to measure the dynamics of gut and oral microbial communities. Each sample was terized using high-throughput sequencing of amplified 16S ribosomal RNA, and the resulting reads were grouped into operational taxonomic units (OTUs) at 97% sequence similarity [19, 20]. After sample quality filtering, we obtained a dataset of 299 gut and 272 saliva samples from Subject A and 180 gut samples from Subject B (Figure 1).

## Results and discussion

### Evidence for long-term, overall community stability

**Figure 2:**
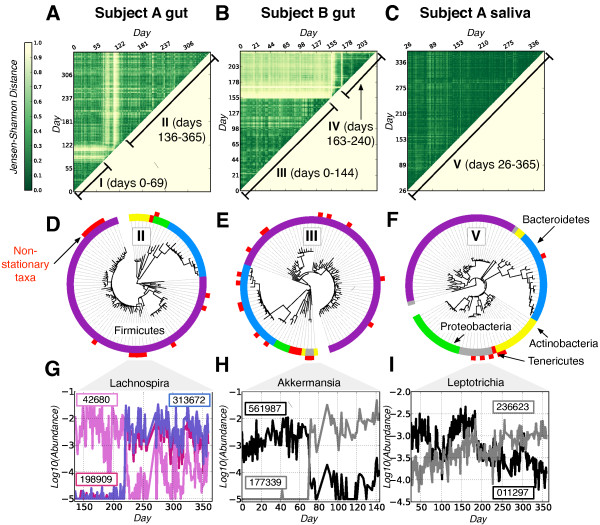
**Stability testing of gut and saliva microbiota time series. (A-C)** Pairwise Jensen Shannon Distances between samples from Subject A’s gut **(A)**, Subject B’s gut **(B)**, and Subject A’s saliva **(C)**. Dark green regions indicate date ranges with similar microbiota. To quantify how stable individual microbial taxa were across the labeled date ranges, we performed the Augmented Dickey Fuller (ADF) test, which evaluated the null hypothesis that a given OTU is non-stationary (that is, the OTU tends to return to an equilibrium value). The majority of tested OTUs were stationary according to the ADF test (88%, 85%, 84%, 79%, and 94% for date ranges I-V, *P* <0.05). **(D-F)** Phylogeny of stationary and non-stationary OTUs. Inner rings denote phyla (the Firmicutes, Bacteroidetes, Proteobacteria, Actinobacteria and Tenericutes are colored purple, blue, green, yellow, and red, respectively). Outer rings are white for stationary OTUs and red for non-stationary ones. Non-stationary taxa clustered phylogenetically for date ranges II **(D)**, III **(E)**, and V **(F)** (*P* <0.05, P-test), supporting the hypothesis that closely-related taxa are more likely to be in competition. **(G-I)** Time series of closely-related, non-stationary OTUs (Greengenes prokMSA ids given in boxes). An artificial abundance floor of 1e-5 was added to improve visibility. Shown are members of the genus *Lachnospira* over date range II **(G)**, the genus *Akkermansia* over date range III **(H)**, and the genus *Leptotrichia* over date range V **(I)**. The summed abundances of the selected *Lachnospira* and *Leptotrichia* are stationary over the given date ranges (*P* <0.05, ADF test).

We initially confirmed the general hypothesis that gut and saliva microbiota are usually stable [6, 8, 9, 18]. First, differences between individuals were much larger than variation within individuals over the course of 1 year (Figure 1A). Second, dynamics within individuals were subdivided into five periods of high overall similarity (Figure 2A-C, regions marked I-V). Third, within these stable periods, median distances between samples rapidly reach an asymptote; these dynamics are consistent with communities whose state is not changing over time (Additional file 3). Fourth, a small subset of highly abundant core taxa can be found within each stable period (Additional file 4). For example, 195 OTUs are found in 95% of Subject A’s saliva microbiota samples over 1 year. These taxa only represent a small minority of the total OTUs detected in Subject A’s saliva, which is consistent with a previous study of human microbiota dynamics [8]. Still, these core OTUs dominate the community and comprise 99.7% of total counted bacteria.

We used an Augmented Dickey-Fuller (ADF) test [22] to quantitatively characterize individual OTU dynamics during periods of apparent overall community stability. The ADF test rejects the existence of a unit root process, such as a random walk, by testing whether time series tend to return to an equilibrium value following fluctuations. Importantly, processes like competition, which leads to the sustained growth of successful taxa and the decline of outcompeted ones, will lead to a failure of the ADF test to reject the null hypothesis. Thus, a significant ADF test suggests a restoring force governing a bacterial species’ dynamics.

We found that most OTUs rejected the ADF null hypothesis, confirming the visual appearance of microbiota stability. The ADF results indicated that between 75% and 88% of bacteria exhibited stationary dynamics during the examined date ranges (Figure 2A-C). Thus, most members of gut and saliva microbial communities may remain stable for periods lasting months. Moreover, we found no correlation between ADF test results and bacterial abundances, indicating that low- and high-abundance taxa are equally likely to be stationary. If OTUs inhabit non-overlapping niches, the observed stationary dynamics may reflect variation in niche sizes due to daily fluctuations in diet and other host factors.

Stability at the level of OTUs, however, does not necessarily indicate a lack of competition and drift. In fact, the remaining 6% to 21% of non-stationary OTUs that fail to reject the ADF null hypothesis may represent competing species in overlapping niches. If genetically similar species are more likely to compete for resources, then these non-stationary OTUs should cluster together phylogenetically. Indeed, we observe support for phylogenetic clustering of non-stationary OTUs in several time ranges (Figure 2D-F) and dynamics consistent with ecological competition (Figure 2G-I). In several cases, species replacement occurred within days (Figure 2G,H). This is surprising because it contrasts with the general stability of OTU abundances and suggests that OTU stability is not simply due to slow microbial dynamics.

In addition to competition between species, competition may occur among populations of bacteria within a single OTU. A recent study of marine bacteria showed significant competition between closely-related populations that would not be distinguishable as distinct OTUs in this study [23]. Thus, our findings support a model in which most OTUs are attracted to an equilibrium level, although ecological competition may occur within OTUs and is sometimes seen among genetically related species.

### Travel and enteric infection are associated with profound community disturbance

Despite the overall evidence for microbiota stability, windows spanning notable host actions and health changes show evidence of broad community disturbance. The first window coincides with Subject A relocating from a major American metropolitan area to the capital of a developing nation in Southeast Asia between days 71 and 122 of the study. This subject was exposed to a novel diet and environment while traveling and had diarrhea on days 80 to 85 and 104 to 113. The second disruptive window accompanies an episode of food poisoning for Subject B, during which the subject tested culture positive for *Salmonella* sp. Consistent with this diagnosis, reads from the *Enterobacteriaceae* (*Salmonella*’s parent family) accounted for a median of 10.1% of daily reads during the diarrheal illness and peaked at 29.3% of reads on day 159 (Additional file 5). Over the entire year, reads from the *Enterobacteriaceae* accounted for a median of 0.004% of each day’s reads. Subject B did not use antibiotics during the diarrheal episode.

**Figure 3:**
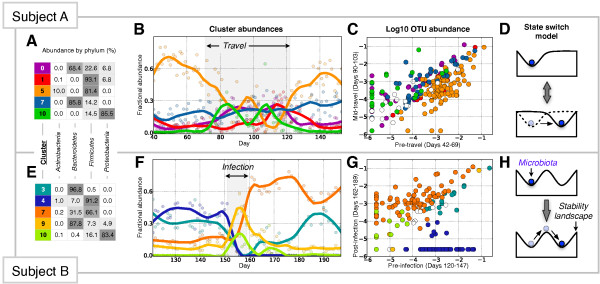
**Dynamics of major OTU clusters across major perturbations.** Highly abundant OTUs were clustered by their dynamics across Subject A’s travel period **(A-D)** and Subject B’s acute enteric infection **(E-H)**. Clusters were produced separately for the two environments; see Methods for more details. **(A,E)** Taxonomic composition of major clusters (fractional abundance exceeds 10% for more than 3 days). **(B,F)** Cluster abundances over time (shaded points) and trend lines (solid) fit using LOESS smoothing, colored using the same scheme as in **(A,E)**. Subject A’s travel abroad (days 71 to 122) and Subject B’s enteric infection (days 151 to 159) are shaded in gray. **(C,G)** Median log_10_(abundance) of OTUs in each cluster before and after perturbation. OTUs are colored by cluster membership, except for uncolored OTUs belonging to clusters not plotted in **(A,E)**. OTU detection limits were set to the minimum fractional abundance observed in each subject’s time series (1e-5.8 for Subject A and 1e-5.6 for Subject B). **(D,H)** Cartoon of microbiome state models, in which microbiota are considered to be balls in a landscape shaped by environmental factors [24]. Subject A’s travel-related microbiota shift is consistent with a model where environmental disturbances cause state changes **(D)**, while Subject B’s infection-related shift is consistent with state transitions caused by direct community perturbations **(H)**.

The two subjects had qualitatively different responses to perturbation. To summarize the broad effects of disturbance across thousands of microbial taxa, we grouped OTUs into a limited number of clusters by their abundance across Subject A’s travel period (Methods). Subject A’s travel strongly perturbed community structure up to the phylum level, coinciding with marked increases and decreases in Bacteroidetes- and Firmicutes-rich clusters, respectively (Figure 3A,B); ultimately, the Bacteroidetes to Firmicutes ratio increased from 0.37 (pre-travel) to 0.71 (mid travel, days 90 to 103; Additional file 6). Despite these changes in species abundance, there was not a large gain or loss of bacterial species (Figure 3C). Of the 352 OTUs present in 95% of samples collected prior to travel, 322 (91%) had non-zero median abundances during the stable travel period. Similarly, of the 359 OTUs present mid-travel, 329 (92%) had non-zero median abundances prior to travel. The emergence of a Proteobacteria-rich cluster of bacteria coincident with diarrhea was a notable exception to the trend of shared species composition before and during travel (Figure 3B), but these species did not persist following return from travel. Thus, dominant post-travel bacterial species were already in place before Subject A’s return.

By contrast, clustering Subject B’s gut microbiota across *Salmonella* infection revealed colonization of new species and the reduction of many commensal species below the sequencing detection limit (Figure 3G). Following infection, OTUs that previously accounted for 44% of pre-infection reads (Cluster 4) made up <1% of reads, while OTUs accounting for only 15% of pre-infection reads (Cluster 7) expanded to represent 65% of post-infection reads (Figure 3F). Enteric infection in Subject B also affected the presence and absence of common pre-infection OTUs. Of the 202 OTUs found in 95% of samples prior to infection, 112 (55%) had a median value of 0 following infection and 28 (13.9%) were not observed again. New OTUs appeared after infection, as 17 (14.7%) of the 116 OTUs found in 95% of post-infection samples had zero median abundance prior to infection. However, these new taxa account for just 1.3% of post-infection reads, indicating that collapses in OTU abundance following enteric infection were primarily compensated for by increased abundances of already present OTUs.

To understand the mechanisms underlying subjects’ departure from their initial microbiota states, we examined the reversibility of each perturbation in the context of recent theories of microbiome ecology. It has been hypothesized that new microbial community states are reached when disturbances either change gut environmental parameters, altering the presence or absence of equilibrium points on a state landscape, or when they alter gut microbiota themselves, shifting communities between fixed equilibria (Figure 3D,H) [9, 13, 25]. Under the environmental disturbance model, reversal of habitat perturbation will restore the original microbiota state. Under the community disturbance model, microbiota can persist in new stable states after the perturbation abates. We investigated which of these models were best supported by community recovery dynamics.

The gut microbiota shift associated with Subject A’s travel reversed upon return home, consistent with the environmental disturbance model of microbiome state transition (Figure 3D). Subject A’s gut microbiota reverted to its pre-travel state in roughly 14 days according to a distance-based analysis (Additional file 7). The reversible state change may have resulted in part from Subject A’s temporary adoption of a regional diet while living abroad. Subject A resumed a normal eating pattern upon returning home, as none of the subject’s tracked dietary variables exhibited significant differences between the months preceding and following travel (q >0.05, Mann–Whitney U test). A microbiota disturbance model driven by regional diet is supported by recent cross-sectional studies, which hypothesize that varying nutritional profiles of non-Western and Western diets promote distinct microbiota in the developed and developing world [26, 27]. Exposure to novel bacteria, including possibly diarrheal pathogens, may have also contributed to Subject A’s altered microbiota. However, elevated beta-diversity scores among samples taken after moving abroad, but before the onset of frank illness, suggests a role for geographic change in altering Subject A’s microbiota (Additional file 7).

In contrast, Subject B’s gut microbiota did not return to its pre-infection state, which is consistent with the community disturbance model (Figure 3H). Indeed, *Salmonella* is known to induce an inflammatory response in the host, which disturbs commensal species and may facilitate pathogen colonization [28]. Subject B’s microbiota persisted in its altered state for the remaining 3 months the subject collected regular fecal samples (Figure 2B).

What forces might enable Subject B’s post-infection gut microbiota to persist? One possibility is that Subject B’s diet changed after infection. However, we did not observe significant changes among Subject B’s dietary variables in the month following infection relative to the month preceding infection (q >0.05, Mann–Whitney U test). An alternative explanation is that the bacterial species lost were replaced by competitors. To test this hypothesis, we reasoned that closely related taxa are likely to share ecological traits [29]. Therefore, we tested whether the bacteria gained (Cluster 7) and lost (Cluster 4) after infection exhibited phylogenetic grouping.

**Figure 4:**
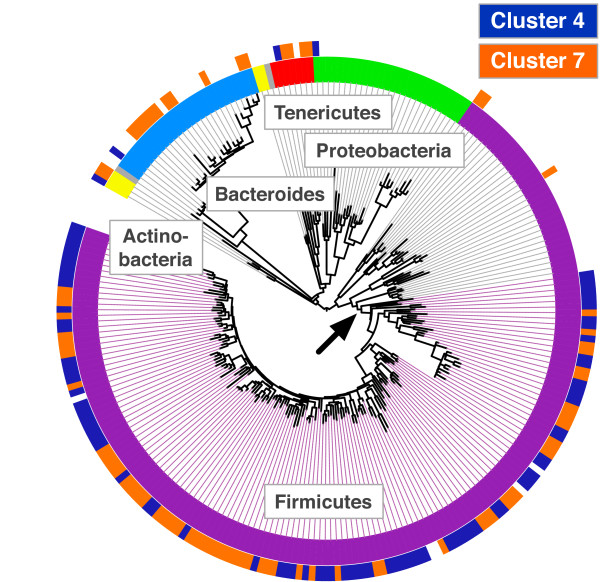
**Phylogenetic evidence for competing gut bacterial taxa.** OTUs clustered by their dynamics across Subject B’s enteric infection (Figure 3) were plotted on a reference phylogeny built using 16S rRNA sequences (Methods). Taxonomic assignments for each OTU are shown on the inner ring and correspond with the color coding from Figure 1. Taxa associated with increasing (Cluster 7, orange) or decreasing (Cluster 4, blue) abundance after infection are indicated on the outer ring. A monophyletic subtree within the Firmicutes (arrowed and shaded) is significantly associated with taxa from the two clusters (*P* <0.001, Fisher’s Exact Test).

The bacterial taxa that expanded in Subject B after infection were indeed closely related to the taxa that were lost, indicating a conservation of function rather than species following infection. We found that taxa from both clusters were primarily associated with a single clade of Firmicutes (Figure 4; *P* <0.001, Fisher’s Exact Test). The increase in Cluster 7 taxa from the Firmicutes subtree after enteric infection (6.9% to 40.1% of reads) nearly mirrored the decline in Cluster 4 taxa from the same subtree (37.2% of reads to 0.04%). Overall, Cluster 4 and 7 taxa from this subtree accounted for 44.1% of reads prior to infection and 40.2% of reads after infection. Thus, we hypothesize that functional stability can be preserved even when compositional stability is lost.

### Lifestyle choices can affect select microbial taxa on daily timescales

We only identified two host experiences that triggered large shifts from stable points, but we found multiple host health and behavior factors that contributed to fluctuations around stable points. Most host factors we tracked behaved randomly over time (Additional file 8), supporting the notion that normal adult lifestyles present microbiota with an idiosyncratic array of daily perturbations. We developed a novel analysis pipeline to identify putative causal links between host factors and microbial time series (Methods). This analysis was designed conservatively and included several steps aimed at filtering out false positive interactions. The pipeline also did not include dates spanning travel or infection events, and it relied on OTU clusters distinct from the ones assembled for the major perturbation analyses. Pipeline development focused on Subject A’s time series because that subject more densely collected metadata than Subject B.

Our analysis identified a subset of gut and salivary OTUs sensitive to host diet and behavior in Subject A (q <0.05, Spearman correlation; Table 1 and Additional files 9, 10, 11). Of the 28 groups of correlations we identified, 25 involved bacterial abundance shifts 1 day after a change in host health or behavior. One notable exception to this pattern is a putative relationship between salivary taxa and host exercise forward in time, which is likely to be a false positive result. For Subject A’s salivary microbiota, we observed flossing to be associated with reduced concentrations of *Streptococcus* species, including the dental pathogen *S. mutans* in the saliva. Flossing has previously been shown to lower *S. mutans* oral load [30]. Unexpectedly, we found body fat and weight negatively correlated with a cluster of oral bacteria. One possible reason for this link is that subject hydration affects body fat measurements on the scales we distributed to subjects, as well as the flow rate, protein levels, and osmolarity of saliva [31]. Changes to these environmental variables may in turn impede the growth of select oral microbes.

**Table 1:** **Significant correlations between Subject A’s metadata and microbiota**

Body site	Lag (days)	Host factor	Representative OTUs (n)	ρ	***P***value	Abun. (%)	Cluster ID	Total OTUs
**Subject A Gut**	0	Stool: Hardness	*Eggerthella*/*Clostridium* (11)	-0.30	1.0E-06	0.2144	10	23
	0	Stool: Time Of Day	*Eggerthella*/*Clostridium* (11)	0.27	7.4E-06	0.2144	10	23
	1	Nutrition: Fiber	*Clostridium* (6)	-0.38	7.4E-06	0.0442	6	9
	1	Nutrition: Fiber	Ruminococcaceae/*F. prausnitzii* (4)	-0.44	1.1E-07	0.3745	8	8
	1	Nutrition: Fiber	*Eggerthella*/*Clostridium* (11)	-0.39	3.3E-06	0.2144	10	23
	1	Nutrition: Fiber	*Ruminococcus*/*R. gnavus*/*Clostridium* (4)	-0.51	2.9E-10	0.5479	51	12
	1	Nutrition: Fiber	*Ruminococcus*/*R. gnavus*/*Clostridium* (5)	-0.51	3.8E-10	0.3495	52	7
	1	Nutrition: Fiber	*Blautia* (3)	-0.38	7.6E-06	0.0346	53	3
	1	Nutrition: Fiber	Bifidobacteriales (13)	0.36	1.6E-05	6.0786	86	13
	1	Nutrition: Fiber	*Coprococcus* (8)	0.44	7.2E-08	4.2192	89	12
	1	Nutrition: Fiber	*Clostridium* (1)	-0.42	4.6E-07	0.0716	111	1
	1	Nutrition: Fiber	*Ruminococcus*/*R. gnavus*/*Clostridium* (6)	-0.44	1.2E-07	2.0690	118	14
	1	Nutrition: Fiber	*Roseburia*/*E. rectale* (30)	0.37	8.4E-06	5.0446	127	40
	1	Food: OrangeJuice	*Clostridium* (1)	0.28	4.7E-06	0.0457	106	2
	1	Food: BreakfastBar	*Ruminococcus*/*R. gnavus*/*Clostridium* (4)	-0.27	6.6E-06	0.5479	51	12
	1	Food: BreakfastBar	*Ruminococcus*/*R. gnavus*/*Clostridium* (5)	-0.40	2.9E-11	0.3495	52	7
	1	Food: BreakfastBar	Bifidobacteriales (13)	0.27	9.5E-06	6.0786	86	13
	1	Food: BreakfastBar	*Clostridium* (1)	-0.43	5.3E-13	0.0716	111	1
	1	Food: Yogurt	Bifidobacteriales (2)	0.45	2.7E-14	0.0069	85	2
	1	Food: Fruits: Fresh	Clostridiales (4)	-0.27	1.1E-05	0.1866	120	9
	1	Food: Fruits: Citrus	Ruminococcaceae/*F. prausnitzii* (4)	0.36	1.7E-09	1.7152	107	4
	1	Food: Soup	Clostridiales (1)	-0.25	3.3E-05	0.0014	62	2
	1	Food: Soup	*Blautia* (21)	-0.26	2.4E-05	3.8126	68	31
	1	Food: Soup: Other	Clostridiales (1)	-0.27	1.3E-05	0.0014	62	2
	1	Food: Soup: Other	*Blautia* (21)	-0.28	4.2E-06	3.8126	68	31
**Subject A Saliva**	-7	Exercise: TookPlace	*S. mutans*/*S. sanguinis* (2)	-0.28	1.6E-05	0.0142	21	2
	1	OralCare: Flossing	*S. mutans*/*S. sanguinis* (2)	-0.30	2.5E-06	0.0142	21	2
	1	Fitness: BodyFat	*Prevotella* (4)	-0.36	1.4E-06	1.5761	35	14

Non-parametric statistics were used to identify metadata variables significantly correlated with clustered OTUs lagged forward or backwards in time (q <0.05, Spearman correlation). Representative taxonomic names from each cluster are shown along with the percentage of overall reads accounted for by each OTU cluster (‘Abundance’). Redundant correlations (for example, yogurt subtypes associated with the same OTU cluster) are shown in Additional file 9, and a full list of taxa associated with each cluster can be found in Additional files 10 and 11. We excluded Subject A’s travel period and Subject B’s post-infection period from metadata correlation analysis. No significant correlations were found among Subject B’s microbiota and metadata.

In Subject A’s gut, fiber-rich foods positively correlated with next-day abundances of clusters comprising more than 15% of total community reads. These clusters were enriched for *Bifidobacteria*, *Roseburia*, and *Eubacterium rectale* species, which previous studies have identified as fiber-sensitive [15, 32–34]. Four *Clostridiales* OTUs, including *Faecalibacterium prausnitzii*, were positively correlated with eating citrus. *F. prausnitzii* is notable for its potential therapeutic role in colitis [35] and is also known to grow on pectin [36], a carbohydrate found in citrus fruit [37]. We also detected a positive correlation between consuming yogurt and *Bifidobacteriales*, which are a common live culture in yogurts.

Using the same gut and salivary OTU clusters tested for metadata interactions, we also explored potential links between gut and saliva microbiota themselves. Healthy humans swallow between 1 L and 1.5 L of saliva daily, making it feasible that oral microbiota are regularly introduced into the digestive tract [38]. Nevertheless, we did not observe any significant temporal correlations between Subject A’s gut and salivary OTU clusters across lag periods ranging from -7 to +7 days (q <0.01, Spearman correlation). Thus, our dataset did not support short-term temporal interactions between gut and salivary microbiota within an individual.

## Conclusions

Despite the relationships inferred above, it is perhaps surprising that given the multiple of tracked host variables, we did not observe more correlations between host behavior and the microbiota. For example, we did not observe extensive links between gut microbiota and variables like sleep, exercise, or mood. These findings suggest that future longitudinal studies of human microbiota will not have to exhaustively control for host behavior, as a wide range of lifestyle attributes are unlikely to broadly disrupt individuals’ microbiota. We note, however, that false negative interactions in this study may have been due to our conservative analysis pipeline, which we biased against inferring false positive correlations. It is also possible that subjects’ self-awareness due to daily tracking skewed our results. To guard against this outcome, we instructed subjects not to deviate from their normal behavior during the study. Moreover, subjects were not aware of their microbial data as the study progressed. Lastly, even though we tracked subjects closely, the range of health and behavioral choices we measured was limited to the individual choices of only two people over 1 year; larger and longer observational studies may cover a broader range of human behaviors and account for temporal effects, like seasonality.

The apparent robustness of human microbiota to many host actions emphasizes the importance of the select host factors that could be linked to microbial dynamics. Notably, two linked host factors, fiber intake and host geography, are thought to influence differences in gut microbiota observed between the Western and developing world. We found that bacteria sensitive to fiber intake could respond to diet shifts within a single day, which is consistent with the hypothesis that fiber plays a major role in shaping gut microbiota [14, 26, 27]. However, a gut microbe commonly found in the developing world and linked to fiber intake, *Prevotella*, did not correlate with subject fiber consumption in our study. In support of the hypothesis that geography plays an important role in shaping gut microbiota [26], we observed that travel to the developing world provided the largest impact to the gut microbiota of one subject over the course of 1 year. Once again, however, this microbiota shift was not linked to changes in the abundance of *Prevotella*. Instead, travel-related changes were associated with abundance changes among already-present bacteria, emphasizing the durability of gut microbial associations with their host [6].

The strength of microbial associations with a host does appear limited though, as we find that a *Salmonella* infection led to persistent declines among more than half of commensal bacterial taxa. This time series represents one of the first longitudinal observations of an enteric bacterial infection in a human adult that includes subject measurements before illness, and it raises key questions for future study. Is post-infection recovery driven by ecological forces such as migration rates and competitive exclusion, or does the host play an active role in rebuilding a stable ecosystem? If the host exerts top-down control of the microbiome, how might it be achieved? Possible mechanisms include pathogen-driven inflammation that ultimately affects tolerance to commensal bacteria [39]. Finally, what are the functional roles carried out by different bacterial taxa leading to their robustness in the face of species turnover, and why might members of certain phyla, like the Firmicutes, be more susceptible to local extinction? Additional studies of human microbiota response to infection are needed to address these questions and should help elucidate the host and ecological forces governing the dynamics of human-associated microbial communities.

## Methods

### Ethical review

We obtained written informed consent from both subjects enrolled in the study. This study was approved by the MIT Committee on the Use of Humans as Experimental Subjects (Study #0903003155) and complied with the Helsinki Declaration.

### Microbial sampling

Subject A collected gut microbiota samples between days 0 and 364 of the study and saliva microbiota samples between days 26 and 364. Subject B primarily collected gut microbiota samples between study days 0 and 252. Gut microbiota were sampled non-invasively using fecal collection. Stool samples were taken in duplicate by coring out feces with inverted sterile 1 mL pipette tips. These tips were then deposited in 15 mL Falcon tubes. Saliva was sampled by 10 s of oral rinsing with 10 mL of sterile phosphate-buffered saline and also stored in 15 mL Falcon tubes. Samples collected at home were stored temporarily at -20°C until transport to the laboratory, where they were then stored in -80°C freezers. Subject A’s samples collected abroad were stored at -20°C, shipped to the United States on dry ice, and then stored at -80°C.

### DNA extraction

We used the QIAamp DNA Stool Mini Kit (Qiagen) and a modified version of its protocol to isolate bacterial DNA from fecal and saliva samples. For stool, we included a bead-beating step at the beginning of DNA extraction, in order to increase cell lysis. First, we used a chilled centrifuge to remove frozen stool cores from the 1 mL pipette tips (30 s at 3,000 g and 4°C). Once samples thawed to 20°C, we added 700 μL of buffer ASL per 100 mg of stool. Next, we used a digital vortex (VWR) and 2 mL of garnet beads (MoBio Laboratories) to break apart stool samples (10 s at 3,000 rpm). We then bead-beat the suspended stool with a Vortex Genie2 (MoBio Laboratories) and 2 mL of 0.1 mm glass beads (MoBio Laboratories) for 10 min at the setting ‘10’, in order to physically lyse cells. Each tube was subsequently heated at 95°C for 6 min to lyse remaining unbroken cells. Afterwards, the Qiagen InhibitEX tablet was added and we followed the QIAamp kit protocol.

For saliva, we initially captured bacterial cells from saliva samples using a 0.22 μm filter (Millipore) and a syringe to apply positive pressure. We placed these filters into 180 μL of lysis buffer (without lysozyme) and bead-beat on a Mini-beadbeater (Biospec products) with 0.1 mm glass beads for 1 min at room temperature and at maximum speed. Next, we added another 180 μL lysis buffer with 40 mg/mL lysozyme and spun for 1 h at 450 rpm and 37°C. Since filtered saliva likely contains fewer PCR inhibitors than stool, we skipped addition of the Qiagen InhibitEX tablet and then followed the remainder of the QIAamp kit protocol.

### DNA sequencing

We used the V4 region of the 16S ribosomal RNA gene subunit to identify bacteria in a culture-independent manner. Extracted DNA was amplified using custom barcoded primers and sequenced with paired-end 100 bp reads on an Illumina GAIIx according to a previously published protocol [19].

### OTU picking

We used the QIIME analysis pipeline (v1.3) to process raw DNA reads into OTU counts [20]. We wrote a Python script to format raw sequence files for input into QIIME. We used the split_libraries_illumina.py QIIME script to initially process reads. To minimize the effects of sequencing errors, we retained only high-quality, full-length reads (max_bad_run_length was set to 0 and the min_per_read_length was assigned to 101). We next used the script parallel_pick_otus_uclust_ref.py to pick OTUs; this script relies on UCLUST [40], which can perform gapped alignments against reference sequences. We used as a reference a set of OTUs assembled at 97% similarity from the Greengenes database [41] (constructed by the nested_gg_workflow.py QiimeUtils script on 4 Feb 2011 [20]). We trimmed the reference FASTA file to span only the 16S region sequenced by our primers.

### Sample quality control

We used pairwise similarity between samples to identify, and subsequently correct or exclude cases of mislabeling or mishandling that may have occurred in our sample processing pipeline. Based on this analysis, we excluded Subject B gut samples from days 229 and 230, which showed an unexpected similarity to those of Subject A. We also excluded a subset of gut samples stored in either ethanol (Subject A gut days 75 and 76) or RNAlater (Subject A gut days 258 to 270) prior to DNA extraction. We chose not to include these samples in our analysis since their storage protocol differed from other samples and could introduce a bias in our results. Finally, samples with unusually low read counts (<10,000) were excluded from further analysis.

### Host metadata

We collected metadata chronicling host health and behavior using iOS devices. We modified a database iOS app (TapForms) to facilitate recording subjects’ daily health and behavior across 13 metadata categories: ailments, bowel movements, daily notes, diet, exercise, fitness, location change, medication, mood, oral hygiene, sleep, urination, and vitamin intake (described in more detail in Additional file 12). At the beginning of the study, subjects were familiarized with the TapForms app and instructed to carry their iOS devices at all times. We asked subjects to record daily health markers and actions relevant to the metadata categories. We then used a custom Python script to parse the TapForms SQL database and generate metadata time series for correlation with OTUs. A template of the TapForms forms used in this study can be downloaded and installed from the GitHub repository [42].

### Augmented Dickey-Fuller (ADF) stationarity testing

We used the ADF test [22] to determine if and when microbial taxa were at equilibrium. We employed the ADF test implemented in the Statsmodels Python module [43]. This method (‘adfuller’) was run on time series mean-centered in log10-space and was called with regression parameters of no constant and no trend (regression = ‘nc’). The number of lags was chosen using the t-statistic (autolag = ‘t-stat’). Test results were compiled for the 100 most abundant OTUs in each microbial community. We chose not to include less-abundant OTUs in these analyses because the dynamics of OTUs near our detection limit are more likely to be noisy. We used the P-test [44] implemented in PyCogent [45] (set to 1,000 permutations and the ‘corrected’ *P* value) to measure the significance of non-stationary OTU clustering on the reference Greengenes 16S rRNA tree.

### Host factor/OTU correlation detection

We constructed a time series analysis pipeline to detect relationships between host metadata and the microbiota. Our pipeline integrated steps to account for numerical artifacts associated with microbiota studies, low abundance OTUs, auto-correlated time series, and to reduce multiple hypothesis testing (Additional file 13). We designed several of these steps to avoid finding spurious correlations between variables, which can commonly occur in time series analysis [46, 47]. To facilitate understanding novel components of our pipeline, we have provided online demonstrations and Python code for time series normalization [48] and detrending auto-correlated time series [49].

#### Normalization

Sequencing-based 16S rRNA surveys are usually normalized by converting OTU sequence counts into fractional abundances for each sample. However, this standard technique leads to what is known as compositional effects [50], and may cause false relationships between OTUs, or between OTUs and metadata. For example, suppose a host switches to a higher fiber diet, which causes fiber-sensitive gut bacteria to multiply, but does not affect fiber-independent OTUs. Standard normalization will suggest the diet shift leads to more fiber-sensitive bacteria and fewer fiber-independent bacteria because the latter group comprises a smaller fraction of the post-diet shift bacterial community. One might then falsely conclude that fiber actively inhibits fiber-independent OTUs when in fact those OTUs do not respond to changes in the nutrient’s availability.

To avoid normalization artifacts when comparing host metadata and microbiota, we developed a novel normalization technique that does not assume sample reads sum to the same fixed value (see subsection below, *Comparison to SparCC*, for more details on how this method differs from our previous work on microbiota correlation). The method we introduce here assumes at least half of the OTUs held in common between two communities do not change in abundance. We then use statistics robust to the effect of outliers to estimate the median OTU fold-change between communities and rescale all OTUs by that value (Additional file 14). Mathematically, we model OTUs in two samples *x* (the observed) and *y* (the reference) as *y*_*i*_ = *m*_*i*_*x*_*i*_ and find the median *m*_*i*_, which we call *m*. We then rescale all *x*_*i*_ by *m*. Unlike the standard normalization, our technique does not infer abundance changes in all OTUs when only a small number actually change. Moreover, since our method does not require that each samples’ reads sum to the same value, we can compare total bacterial load between samples.

Our regression technique is implemented as follows. First, we normalized time points in the standard manner so that all fractional OTU abundances each day sum to 1. Second, we restricted our analysis to a subset of highly-abundant OTUs, since regularly undetected OTUs will have a zero or undefined *m*_*i*_. We then sorted OTUs by abundance and selected the first set of OTUs that accounted for 90% of median daily reads. Third, we randomly chose time points to normalize. We normalized each time point to a reference community (rather than a single time point), to minimize the effects of anomalous time points during normalization. We did not use the same reference community for each time point since multiple microbiota states may exist in a single time series (for example, Subject B before and after diarrheal infection). Rather, we computed a reference for each sample based on other time points with similar community structure. We used a weighted median across all time points to compute reference OTU values, where we set time point weights to be (1 - *j*)^2^ and *j* was the pairwise JSD score to the sample being normalized. Our OTU abundance data appeared heteroscedastic, meaning that the variance of more abundant OTUs was higher than the variance of less abundant OTUs. We applied a common solution to this problem, which was to solve for *m*_*i*_ in log-space. Fourth, we discarded time points with an uncertain estimate of *m* (median(|*y*_*i*_ - *mx*_*i*_|) > 0.4).

Because no gold standard dataset matches sequencing-base 16S surveys to overall bacterial load in the human gut, we used simulated data to test our normalization scheme. We modeled synthetic microbial communities on microbiota observed in our experiments. Each OTU in our synthetic community behaved according to an Ornstein-Uhlenbeck (OU) process, which can be thought of as a random-walk modified to mean-revert over time. We simulated an OU process using the following function [51], where *S*_*i*_ is OTU abundance at time *i*, λ describes how quickly the process returns to the mean, μ is the mean, σ the average magnitude of fluctuations, and δ is the time between simulation steps (we set to 1):


We calculated maximum likelihood OU parameters for 3,383 OTUs drawn from Subject B’s gut time series using the following system of equations [51]:


To test our normalization technique in the face of microbiota perturbations, we simulated two OU processes for each OTU: one calibrated using Subject B’s pre-infection samples and one using post-infection samples. We joined these processes (each with 50 time points) into a single time series (100 time points). Lastly, we simulated daily microbiota surveys by randomly sampling OTUs according to their fractional abundance at each time point. We randomly chose total read counts from the set of read counts observed in Subject B’s microbiota time series.

Our robust regression accurately normalized the simulated time series. We evaluated our method by first tracking changes in bacterial load over time within our simulated communities. We compared these changes to bacterial load predictions from normalized time series of synthetic sequencing runs. We note that the standard normalization approach cannot infer bacterial load changes, since it predicts each samples’ OTU abundances sum to the same value. Over four separate synthetic datasets, the Spearman correlation between simulated bacterial loads and our inferred bacterial loads was never less than 0.58 (all *P* values ≤1.37e-10; Additional file 15), even after accounting for the autocorrelated nature of total bacterial load (see section below on *Autocorrelation elimination*).

#### Comparison to SparCC

Surveys of 16S rRNA are usually treated as fractional abundances, rather than absolute ones. This traditional approach leads to read totals that sum to 1, meaning fractions cannot change independently of each other; this in turn could lead to false relationships between OTUs, or between OTUs and metadata [52]. We have previously developed a method, termed SparCC, for inferring correlations between OTU from genomic survey data [50]. However, SparCC applies to independent samples, while this study is concerned with autocorrelated time series. Moreover, in this study we are interested in inferring correlations between OTUs and metadata, whereas SparCC focuses solely on inter-OTU correlations. We therefore introduced here a new method for normalizing 16S rRNA time series, as well as finding metadata-OTU correlations.

#### OTU filtering

We only tested relationships between common OTUs (present in at least half of a given period’s samples) and host metadata. Focusing on common OTUs increased the likelihood we detected true interactions, since we could analyze shifts in bacterial abundance and not simply OTU presence or absence. Moreover, filtering out OTUs reduced the total number of statistical tests we performed and thus reduced the burden of multiple hypothesis testing. After filtering, 750 OTUs, 621 OTUs, and 289 OTUs remained from Subject A’s gut, Subject B’s gut, and Subject A’s salivary microbiota time series, respectively.

#### Autocorrelation elimination

Autocorrelated processes occur when measurements taken at one time point are correlated with measurements at previous or future time points. For example, subjects’ weights in this study are autocorrelated, as their weight on a given day is likely to be highly similar to their weight the previous day. This poses a challenge for finding statistical relationships between host metadata and their microbiota, because it is well-known in time series analysis that cross-correlations between autocorrelated variables have unreliable *P* values [46, 47]. To avoid this problem, we fitted time series models to each variable and computed cross-correlations on the differences (residuals) between modeled trends and the observed data [47]. For the microbial time series, we use the R (ver. 2.15.1) ‘forecast’ package to fit standard time series models known as autoregressive integrated moving average (ARIMA) models [53]. ARIMA models are commonly used tools in econometrics and time series analysis to model longitudinal data [46]. We fit ARIMA parameters using the ‘auto.arima’ function with a maximum *p* of 2, a maximum *q* of 2, and a *d* of either 0 or 1. We chose which value of *d* to use by minimizing a common measure of model complexity (Bayesian information criterion). We applied a similar procedure to the host metadata, except in the case of variables whose behavior appeared binary (that is, in at least 75% of the time series, the variable had a value of zero). Because ARIMA models may not be appropriate for non-continuous time series [54], we used a logistic regression model designed for binary longitudinal data (the R ‘bild’ package [55]). We calculated two kinds of serial dependence models (first-order and second-order) for each metadata variable and again picked the one that minimized a measure of model complexity (the Akaike information criterion). In all cases, if the autocorrelation of the residual time series was higher than the autocorrelation of the original time series itself, we discarded the residual series and worked only on the original data.

#### Clustering

To further reduce the number of tested microbial and metadata interactions, we clustered OTUs sharing similar temporal dynamics. We computed pairwise distances between OTUs as 1-ρ, where ρ was the Spearman correlation (‘rcorr’ function in the R ‘hmisc’ package [56]) between the OTUs’ time series. We rounded negative distances up to 0. We next passed the OTU distance matrix to the ‘linkage’ function in the SciPy [57] (ver. 10.1) hierarchical clustering package (scipy.cluster.hierarchy). We used the ‘weighted’ linkage method to compute OTU clustering. Cluster assignments were retrieved using the ‘fcluster’ function with the clustering criterion set to ‘distance’ and a clustering threshold of 80% of the maximum distance between nodes in the linkage matrix. This pipeline produced 138 clusters for Subject A’s gut time series, 90 clusters for Subject B’s gut time series, and 46 clusters for Subject A’s salivary time series (Additional file 16). We modeled cluster dynamics using the median OTU value at each time point over all the OTUs within the cluster. Lastly, to again guard against autocorrelations in the cluster time series, we fit ARIMA models to each cluster and computed residual time series.

#### Correlation

We used rank-based non-parametric statistics to detect correlations between time series of detrended OTU clusters and detrended metadata. We lagged the cluster time series between -7 and +7 days, relative to the metadata, and computed Spearman correlations again using the ‘rcorr’ function in the R ‘hmisc’ package. Microbial or metadata variables with high autocorrelation (*P* <0.01) were excluded from analysis. We estimated false discovery rates separately for a given lag and body site (‘fdrtool’ R package [58]). As a final check for spurious cross-correlations [47], we excluded interactions that when regressed against each other, exhibited auto-correlated errors (*P* <0.01, Durbin-Watson test [59]).

### Disturbance analyses

To simplify our analysis of how OTUs responded to prolonged travel abroad or enteric infection, we constructed a clustering pipeline similar to the one used in our host-factor/microbiota correlation testing. We inputted standardly normalized time series into this pipeline because our robust regression-based normalization routine could not confidently infer scaling factors during both Subject A and B’s diarrheal illnesses. We also used a slightly more permissive clustering threshold than the previous section (90% of the maximum distance between nodes in the linkage matrix) because we wanted to study broad bacterial trends and not more minor OTU dynamical patterns. This clustering pipeline yielded 11 OTU clusters for both subjects’ time series (Additional file 17).

We used Fischer’s exact test (SciPy.stats) to determine if clustered OTUs shared significant phylogenetic similarity. We used the Greengenes 16S tree from OTU picking as our reference phylogeny and the PyCogent library [45] to analyze this tree. Closely-related taxa with similar temporal dynamics could reflect sequencing errors associated with a single strain; this artifact could in turn cause falsely-significant phylogenetic grouping. To control for such errors, we collapsed subtrees of OTUs sharing the same cluster assignment and leaf pairwise distances less than 0.2 down to a single leaf. We then used the collapsed reference tree to construct a 2X2 contingency table with rows counting how many OTUs were part of, or excluded from, a given set of clusters and columns counting how many OTUs were within, or outside of, a given subtree.

### Data availability

The read data for all samples have been deposited in the European Bioinformatics Institute (EBI) European Nucleotide Archive (ENA) under the nucleotide accession number ERP006059. Subject A’s nutritional metadata (that is, estimated daily intake of calories, total fat, saturated fat, cholesterol, protein, sodium, carbohydrates, fiber, sugars, and calcium) are provided under the group accession number SAMEG179160 and can also be found in Additional file 18. For other metadata requests, please contact the corresponding author.

## Electronic supplementary material

Additional file 1: **Host metadata categories.** We processed host daily records into 349 variables grouped into the categories: ailments, bowel movements, exercise, fitness, specific food intake (parsed by a text-mining algorithm modeled on a food frequency questionnaire), location, medication, mood, nutrition (measured via the CalorieKing database), oral hygiene, sleep, urination, and vitamin supplementation. (XLS 14 KB)

Additional file 2: **Subject demographic information.** Subjects were unrelated men who volunteered for extensive personal tracking. (XLSX 36 KB)

Additional file 3: **Microbiota similarity over time measured with the Jensen-Shannon Distance (JSD).** (A-C) Pairwise JSD distances between Subject A gut samples (A), Subject B gut samples (B), and Subject A saliva samples (C). (D-K) Median pairwise JSD as a function of sample temporal distance (blue points). The median value for each curve is shown as a solid red line. Asymptotic curves, which appear to converge on the solid red lines, are consistent with the notion of a stable microbiota over a given date range (D-F, H, K). Notably, pairwise JSD curves spanning distinct stable periods do not exhibit asymptotic behavior (G), or have relatively high asymptotes (J). (PDF 47 KB)

Additional file 4: **Highly abundant OTUs are also persistent.** Curves show the fraction of total reads (blue) and the fraction of total OTUs (green) accounted for by OTUs present in at least a given fraction of samples. Curves made using (A) Subject A gut samples from days 0 to 69 and 136 to 364, (B) Subject B gut samples from days 0 to 144, and (C) all Subject A saliva samples. (PDF 66 KB)

Additional file 5: **Fractional abundance of** ***Enterobacteriaceae*** **over time in Subject B’s gut.** Each colored point represents the abundance of *Enterobacteriaceae* on a given date. Subject B suffered from a diarrheal illness from days 151 to 159 of the study, during which he was culture-positive for *Salmonella*. The *Enterobacteriaceae*, the parent family of *Salmonella*, account for a median of 0.004% of daily reads over the entire time series. During days 151 to 159, this family comprises a median of 10.1% of each day’s reads and peaks at 29.3% of reads on day 159. (PDF 125 KB)

Additional file 6: **Bacteroidetes to Firmicutes ratio over time in Subject A’s gut.** Subject A’s prolonged travel abroad shown in gray (days 71 to 122). The median Bacteroidetes/Firmicutes ratio in Subject A’s gut was 0.37 for days <70, 0.71 for days 90 to 103, and 0.38 for days >122. (PDF 333 KB)

Additional file 7: **Gut microbiota shifts across travel.** Plotted over time is the Jensen-Shannon Distance (JSD) between Subject A gut microbiota and the median gut microbial community when the subject lived in the United States. Subject A left the United States on day 70 and returned on day 122 (travel period shaded in gray); he suffered from diarrheal illnesses between days 80 and 85 and days 104 and 113 (red shading). The red dashed line denotes the median JSD between domestic gut microbiota samples and the median domestic gut microbiota. The JSD increase after arriving abroad, but before the first diarrheal illness (days 71 to 79) argues that travel abroad was sufficient to alter Subject A’s gut microbiota. The JSD declines below the red median JSD line on day 136, suggesting that recovery of gut microbiota from travel required 14 days. (PDF 1 MB)

Additional file 8: **Statistics of host metadata dynamics.** We measured day-to-day variability of host factors using the 1-day autocorrelation, which quantifies the correlation between a variable and its value the following day. (A) Autocorrelation of metadata variables tracked in Subjects A and B. Variables are colored by metadata category. Variables whose autocorrelation is only defined for one subject are shown using single-axis scatter plots. Most tracked host factors behaved randomly over time: the median autocorrelation across host factors was 0.14 in Subject A and 0.06 in Subject B. Exceptions to this trend were subject location, weight and body fat, which had autocorrelations >0.4 in both subjects. (B) Scatter plots of day-to-day variation among host factors with varying autocorrelation. Each point represents metadata value on a given day (t: x-axis) and the following day (t + 1: y-axis). (PDF 320 KB)

Additional file 9: **All significant correlations (q <0.05) between subject metadata and microbiota.** Table includes ‘redundant’ correlations involving similar food items and the same OTU cluster (for example, Food:Yogurt and Food:Yogurt:Non-Activia are both correlated with OTU cluster 84). A non-redundant list of correlations is presented in Table 1. (XLS 34 KB)

Additional file 10: **Taxonomy of Subject A saliva OTUs correlated with host metadata.** Shown are oral bacterial taxa assigned to bacterial clusters that have significant correlations with host health or lifestyle (Table 1). Clusters were defined using hierarchical clustering (Additional file 16) of bacteria with similar temporal dynamics. Taxonomic IDs and Latin names are drawn from the Greengenes database. (XLS 14 KB)

Additional file 11: **Taxonomy of Subject A gut OTUs correlated with host metadata.** Shown are gut bacterial taxa assigned to bacterial clusters that have significant correlations with host health or lifestyle (Table 1). Clusters were defined using hierarchical clustering (Additional file 16) of bacteria with similar temporal dynamics. Taxonomic IDs and Latin names are drawn from the Greengenes database. (XLS 48 KB)

Additional file 12: **Description of host metadata categories.** (PDF 47 KB)

Additional file 13: **Flowchart of time series analysis pipeline for detecting OTU-metadata correlations.** We implemented normalization, low-abundance OTU filtering, and autocorrelation elimination steps to reduce the likelihood of inferring spurious correlations. We also used OTU filtering and clustering to reduce the number of hypothetical OTU-metadata interactions tested. (PDF 66 KB)

Additional file 14: **Regression-based normalization example.** (A) A toy community with five OTUs (blue dots) sampled on 2 different days is used to illustrate our normalization scheme. The Day 2 sample is sequenced twice as deeply as the Day 1 sample. Moreover, the community is unchanged across these days, except for one OTU that decreases by 90% on Day 2 (arrowed). Our normalization technique uses regression to infer relative differences in total bacterial abundance between samples. We use median-based line-fitting (blue line), which is robust to outliers and whose slope reflects the two-fold difference in sequencing depth. A standard least-squares regression through the origin is affected by the OTU with sharply decreased abundance. (B) Day 2 OTUs rescaled by the robust regression scaling factor are unchanged relative to Day 1 (blue dots). By contrast, rescaling Day 2’s OTUs with standard techniques (Day 2 OTU levels sum to Day 1 OTU levels; green dots) causes artifactual day-to-day changes among four OTUs (arrowed). (PDF 125 KB)

Additional file 15: **Results of testing normalization with four simulated datasets.** (A) We simulated bacterial communities over time using Ornstein-Uhlenbeck processes fit to observed gut bacterial dynamics in Subject B. Total bacterial load among the synthetic communities varied over time (blue line; mean-centered on 1). We simulated 16S sequencing runs using the synthetic time series, and input the runs into our robust regression-based normalization. Bacterial load estimated from the normalized communities (red line) closely tracked the simulated bacterial load, suggesting our normalization scheme is accurate. (B) Even after accounting for autocorrelations in bacterial load over time (see Methods section on *Autocorrelation elimination*), we observed significant correlations between the simulated and inferred bacterial loads. (PDF 333 KB)

Additional file 16: **Hierarchical clustering of detrended OTUs.** Matrices show pairwise Spearman correlations between detrended OTU time series. Red points correspond with OTU pairs sharing more positive correlations, and blue points correspond with OTU pairs sharing more negative pairwise correlations. We used correlation matrices to perform hierarchical clustering. Clustered OTUs are segregated on this hierarchy by line color. Clustering yielded 138, 90, and 46 OTU clusters for Subject A’s gut, Subject B’s gut, and Subject A’s saliva time series, respectively. These clusters were ultimately tested against host factors to detect microbiota-lifestyle interactions (Table 1). (PDF 1 MB)

Additional file 17: **Hierarchical clustering of OTUs across disturbances.** Matrices show pairwise Spearman correlations between OTUs tracked before and after Subject A’s prolonged travel abroad or Subject B’s acute enteric infection. Red points correspond with OTU pairs sharing more positive correlations, and blue points correspond with OTU pairs sharing more negative pairwise correlations. We used correlation matrices to perform hierarchical clustering. Clustered OTUs are segregated on this hierarchy by line color. Clustering yielded 11 OTU clusters for both time series. Clusters here were used to analyze subjects’ gut microbiota response to large perturbations (Figures 3 and 4). (PDF 320 KB)

Additional file 18: **Sample and nutritional metadata.** Metadata are provided for nucleotide sequences deposited on the EBI/ENA database under accession number ERP006059. These metadata include Subject A’s nutritional data, provided for the day preceding each sample. (CSV 914 KB)

## Abbreviations

ADF: Augmented Dickey-Fuller
ARIMA: autoregressive integrated moving average
EBI: European Bioinformatics Institute
ENA: European Nucleotide Archive
GAIIx: Genome Analyzer IIx
JSD: Jensen-Shannon distance
LOESS: LOcal regrESSion
OTU: operational taxonomic unit
OU: Ornstein-Uhlenbeck
QIIME: Quantitative Insights into Microbial Ecology
rRNA: ribosomal RNA
SQL: Structured query language.

## References

[1] Cho I, Yamanishi S, Cox L, Methé BA, Zavadil J, Li K, Gao Z, Mahana D, Raju K, Teitler I, Li H, Alekseyenko AV, Blaser MJ (2012). Antibiotics in early life alter the murine colonic microbiome and adiposity. Nature.

[2] Turnbaugh PJ, Ridaura VK, Faith JJ, Rey FE, Knight R, Gordon JI (2009). The effect of diet on the human gut microbiome: a metagenomic analysis in humanized gnotobiotic mice. Sci Transl Med.

[3] Buffie CG, Jarchum I, Equinda M, Lipuma L, Gobourne A, Viale A, Ubeda C, Xavier J, Pamer EG (2012). Profound alterations of intestinal microbiota following a single dose of clindamycin results in sustained susceptibility to Clostridium difficile-induced colitis. Infect Immun.

[4] Turnbaugh PJ, Ley RE, Mahowald MA, Magrini V, Mardis ER, Gordon JI (2006). An obesity-associated gut microbiome with increased capacity for energy harvest. Nature.

[5] Devkota S, Wang Y, Musch MW, Leone V, Fehlner-Peach H, Nadimpalli A, Antonopoulos DA, Jabri B, Chang EB (2012). Dietary-fat-induced taurocholic acid promotes pathobiont expansion and colitis in Il10-/- mice. Nature.

[6] Faith JJ, Guruge JL, Charbonneau M, Subramanian S, Seedorf H, Goodman AL, Clemente JC, Knight R, Heath AC, Leibel RL, Rosenbaum M, Gordon JI (2013). The long-term stability of the human gut microbiota. Science.

[7] Zoetendal EG, Akkermans AD, de Vos WM (1998). Temperature gradient gel electrophoresis analysis of 16S rRNA from human fecal samples reveals stable and host-specific communities of active bacteria. Appl Environ Microbiol.

[8] Caporaso JG, Lauber CL, Costello EK, Berg-Lyons D, González A, Stombaugh J, Knights D, Gajer P, Ravel J, Fierer N, Gordon JI, Knight R (2011). Moving pictures of the human microbiome. Genome Biol.

[9] Dethlefsen L, Relman DA (2011). Incomplete recovery and individualized responses of the human distal gut microbiota to repeated antibiotic perturbation. Proc Natl Acad Sci U S A.

[10] Dethlefsen L, Huse S, Sogin ML, Relman DA (2008). The pervasive effects of an antibiotic on the human gut microbiota, as revealed by deep 16S rRNA sequencing. PLoS Biol.

[11] Jakobsson HE, Jernberg C, Andersson AF, Sjölund-Karlsson M, Jansson JK, Engstrand L (2010). Short-term antibiotic treatment has differing long-term impacts on the human throat and gut microbiome. PLoS One.

[12] Ley RE, Turnbaugh PJ, Klein S, Gordon JI (2006). Microbial ecology: Human gut microbes associated with obesity. Nature.

[13] Hartman AL, Lough DM, Barupal DK, Fiehn O, Fishbein T, Zasloff M, Eisen JA (2009). Human gut microbiome adopts an alternative state following small bowel transplantation. Proc Natl Acad Sci U S A.

[14] Wu GD, Chen J, Hoffmann C, Bittinger K, Chen Y-Y, Keilbaugh SA, Bewtra M, Knights D, Walters WA, Knight R, Sinha R, Gilroy E, Gupta K, Baldassano R, Nessel L, Li H, Bushman FD, Lewis JD (2011). Linking long-term dietary patterns with gut microbial enterotypes. Science.

[15] David LA, Maurice CF, Carmody RN, Gootenberg DB, Button JE, Wolfe BE, Ling AV, Devlin AS, Varma Y, Fischbach MA, Biddinger SB, Dutton RJ, Turnbaugh PJ (2014). Diet rapidly and reproducibly alters the human gut microbiome. Nature.

[16] Gajer P, Brotman RM, Bai G, Sakamoto J, Schütte UME, Zhong X, Koenig SSK, Fu L, Ma ZS, Zhou X, Abdo Z, Forney LJ, Ravel J (2012). Temporal dynamics of the human vaginal microbiota. Sci Transl Med.

[17] Koenig JE, Spor A, Scalfone N, Fricker AD, Stombaugh J, Knight R, Angenent LT, Ley RE (2011). Succession of microbial consortia in the developing infant gut microbiome. Proc Natl Acad Sci U S A.

[18] Costello EK, Lauber CL, Hamady M, Fierer N, Gordon JI, Knight R (2009). Bacterial community variation in human body habitats across space and time. Science.

[19] Caporaso JG, Lauber CL, Walters WA, Berg-Lyons D, Lozupone CA, Turnbaugh PJ, Fierer N, Knight R (2011). Global patterns of 16S rRNA diversity at a depth of millions of sequences per sample. Proc Natl Acad Sci U S A.

[20] Caporaso JG, Kuczynski J, Stombaugh J, Bittinger K, Bushman FD, Costello EK, Fierer N, Peña AG, Goodrich JK, Gordon JI, Huttley GA, Kelley ST, Knights D, Koenig JE, Ley RE, Lozupone CA, McDonald D, Muegge BD, Pirrung M, Reeder J, Sevinsky JR, Turnbaugh PJ, Walters WA, Widmann J, Yatsunenko T, Zaneveld J, Knight R (2010). QIIME allows analysis of high-throughput community sequencing data. Nat Methods.

[21] Heer J, Kong N, Agrawala M (2009). Sizing the horizon: The effects of chart size and layering on the graphical perception of time series visualizations. Proceedings of the 27th international conference on Human factors in computing systems. Volume CHI ‘09.

[22] Said SE, Dickey DA (1984). Testing for unit roots in autoregressive-moving average models of unknown order. Biometrika.

[23] Cordero OX, Ventouras L-A, DeLong EF, Polz MF (2012). Public good dynamics drive evolution of iron acquisition strategies in natural bacterioplankton populations. Proc Natl Acad Sci U S A.

[24] Beisner B, Haydon DT, Cuddington K (2003). Alternative stable states in ecology. Front Ecol Environ.

[25] Costello EK, Stagaman K, Dethlefsen L, Bohannan BJM, Relman DA (2012). The application of ecological theory toward an understanding of the human microbiome. Science.

[26] Yatsunenko T, Rey FE, Manary MJ, Trehan I, Dominguez-Bello MG, Contreras M, Magris M, Hidalgo G, Baldassano RN, Anokhin AP, Heath AC, Warner B, Reeder J, Kuczynski J, Caporaso JG, Lozupone CA, Lauber C, Clemente JC, Knights D, Knight R, Gordon JI (2012). Human gut microbiome viewed across age and geography. Nature.

[27] De Filippo C, Cavalieri D, Di Paola M, Ramazzotti M, Poullet JB, Massart S, Collini S, Pieraccini G, Lionetti P (2010). Impact of diet in shaping gut microbiota revealed by a comparative study in children from Europe and rural Africa. Proc Natl Acad Sci U S A.

[28] Stecher B, Robbiani R, Walker AW, Westendorf AM, Barthel M, Kremer M, Chaffron S, Macpherson AJ, Buer J, Parkhill J, Dougan G, Mering von C, Hardt W-D (2007). Salmonella enterica serovar typhimurium exploits inflammation to compete with the intestinal microbiota. PLoS Biol.

[29] Horner-Devine MC, Bohannan BJM (2006). Phylogenetic clustering and overdispersion in bacterial communities. Ecology.

[30] Corby PMA, Biesbrock A, Bartizek R, Corby AL, Monteverde R, Ceschin R, Bretz WA (2008). Treatment outcomes of dental flossing in twins: molecular analysis of the interproximal microflora. J Periodontol.

[31] Walsh NP, Montague JC, Callow N, Rowlands AV (2004). Saliva flow rate, total protein concentration and osmolality as potential markers of whole body hydration status during progressive acute dehydration in humans. Arch Oral Biol.

[32] Walker AW, Ince J, Duncan SH, Webster LM, Holtrop G, Ze X, Brown D, Stares MD, Scott P, Bergerat A, Louis P, McIntosh F, Johnstone AM, Lobley GE, Parkhill J, Flint HJ (2010). Dominant and diet-responsive groups of bacteria within the human colonic microbiota. ISME J.

[33] Duncan SH, Belenguer A, Holtrop G, Johnstone AM, Flint HJ, Lobley GE (2007). Reduced dietary intake of carbohydrates by obese subjects results in decreased concentrations of butyrate and butyrate-producing bacteria in feces. Appl Environ Microbiol.

[34] Gibson GR, Beatty ER, Wang X, Cummings JH (1995). Selective stimulation of bifidobacteria in the human colon by oligofructose and inulin. Gastroenterology.

[35] Sokol H, Pigneur B, Watterlot L, Lakhdari O, Bermúdez-Humarán LG, Gratadoux J-J, Blugeon S, Bridonneau C, Furet J-P, Corthier G, Grangette C, Vasquez N, Pochart P, Trugnan G, Thomas G, Blottière HM, Doré J, Marteau P, Seksik P, Langella P (2008). Faecalibacterium prausnitzii is an anti-inflammatory commensal bacterium identified by gut microbiota analysis of Crohn disease patients. Proc Natl Acad Sci U S A.

[36] Lopez-Siles M, Khan TM, Duncan SH, Harmsen HJM, Garcia-Gil LJ, Flint HJ (2012). Cultured representatives of two major phylogroups of human colonic Faecalibacterium prausnitzii can utilize pectin, uronic acids, and host-derived substrates for growth. Appl Environ Microbiol.

[37] Baker RA (1997). Reassessment of some fruit and vegetable pectin levels. J Food Sci.

[38] Humphrey SP, Williamson RT (2001). A review of saliva: normal composition, flow, and function. J Prosthet Dent.

[39] Hand TWT, Santos Dos LML, Bouladoux NN, Molloy MJM, Pagán AJA, Pepper MM, Maynard CLC, Elson COC, Belkaid YY (2012). Acute gastrointestinal infection induces long-lived microbiota-specific T cell responses. Science.

[40] Edgar RC (2010). Search and clustering orders of magnitude faster than BLAST. Bioinformatics.

[41] DeSantis TZ, Hugenholtz P, Larsen N, Rojas M, Brodie EL, Keller K, Huber T, Dalevi D, Hu P, Andersen GL (2006). Greengenes, a chimera-checked 16S rRNA gene database and workbench compatible with ARB. Appl Environ Microbiol.

[42] **Tapforms forms used in longitudinal diet study** [http://github.com/ladavid/mit_tapforms]

[43] Seabold S, Perktold J (2010). Statsmodels: Econometric and statistical modeling with python. Proceedings of the 9th Python in Science Conference.

[44] Martin AP (2002). Phylogenetic approaches for describing and comparing the diversity of microbial communities. Appl Environ Microbiol.

[45] Knight R, Maxwell P, Birmingham A, Carnes J, Caporaso JG, Easton BC, Eaton M, Hamady M, Lindsay H, Liu Z, Lozupone C, McDonald D, Robeson M, Sammut R, Smit S, Wakefield MJ, Widmann J, Wikman S, Wilson S, Ying H, Huttley GA (2007). PyCogent: a toolkit for making sense from sequence. Genome Biol.

[46] Chatfield C (2003). The Analysis of Time Series: an Introduction.

[47] Granger CWJ, Newbold P (1974). Spurious regressions in econometrics. J Econ.

[48] **Normalizing microbiota time-series data** [http://nbviewer.ipython.org/github/ladavid/mit_timeseries/blob/master/NormalizeDemo.ipynb]

[49] **Detrending auto-correlated data** [http://nbviewer.ipython.org/github/ladavid/mit_timeseries/blob/master/DetrendDemo.ipynb]

[50] Friedman J, Alm EJ (2012). Inferring correlation networks from genomic survey data. PLoS Comput Biol.

[51] **Calibrating the Ornstein-Uhlenbeck (Vasicek) model** [http://www.sitmo.com/article/calibrating-the-ornstein-uhlenbeck-model/]

[52] Aitchison J (2003). The Statistical Analysis of Compositional Data.

[53] Hyndman RJ, Khandakar Y (2008). Automatic Time Series Forecasting: The forecast Package for R. J Stat Softw.

[54] Cardinal M, Roy R, Lambert J (1999). On the application of integer-valued time series models for the analysis of disease incidence. Stat Med.

[55] Gonçalves MH, Cabral MS (2012). The R Package bild for the Analysis of Binary Longitudinal Data. J Stat Softw.

[56] **Hmisc** [http://cran.r-project.org/web/packages/Hmisc/index.html]

[57] Jones E, Oliphant T, Peterson P: **SciPy: Open source scientific tools for Python.** [http://www.scipy.org]

[58] Strimmer K (2008). fdrtool: a versatile R package for estimating local and tail area-based false discovery rates. Bioinformatics.

[59] Zeileis A, Hothorn T (2002). Diagnostic checking in regression relationships. R news.

