# Erratum to: Host lifestyle affects human microbiota on daily timescales

**DOI:** 10.1186/s13059-016-0988-y

**Published:** 2016-05-31

**Authors:** Lawrence A. David, Arne C. Materna, Jonathan Friedman, Maria I. C. Baptista, Matthew C. Blackburn, Allison Perrotta, Susan E. Erdman, Eric J. Alm

**Affiliations:** Society of Fellows, Harvard University, Cambridge, MA 02138 USA; FAS Center for Systems Biology, Harvard University, Cambridge, MA 02138 USA; QIAGEN Aarhus A/S, Silkeborgvej 2, Aarhus C, 8000 Denmark; Computational & Systems Biology, Massachusetts Institute of Technology, Cambridge, MA 02139 USA; Koch Institute for Integrative Cancer Research, Massachusetts Institute of Technology, Cambridge, MA 02139 USA; Institute of Bioengineering, School of Engineering, École Polytechnique Fédérale de Lausanne, Lausanne, CH-1015 Switzerland; Department of Civil & Environmental Engineering, Massachusetts Institute of Technology, Cambridge, MA 02139 USA; Division of Comparative Medicine, Massachusetts Institute of Technology, Cambridge, MA 02139 USA; Department of Biological Engineering, Massachusetts Institute of Technology, Cambridge, MA 02139 USA; The Broad Institute of MIT and Harvard, Cambridge, MA 02139 USA; Present address: Molecular Genetics & Microbiology and Center for Genomic & Computational Biology, Duke University, Durham, NC 27708 USA

As a result of a production error during the type-setting of the final version of the article [1], a number of additional files were incorrectly published, with the files not matching the Additional Files legends. All additional files for this article are republished below in the correct order. The publisher apologizes for the error and any confusion caused.

## Additional files

Additional file 1: Host metadata categories. We processed host daily records into 349 variables grouped into the categories: ailments, bowel movements, exercise, fitness, specific food intake (parsed by a text-mining algorithm modeled on a food frequency questionnaire), location, medication, mood, nutrition (measured via the CalorieKing database), oral hygiene, sleep, urination, and vitamin supplementation. (XLS 82 kb) Additional file 2: Subject demographic information. Subjects were unrelated men who volunteered for extensive personal tracking. (XLSX 35 kb) Additional file 3: Microbiota similarity over time measured with the Jensen-Shannon Distance (JSD). (A-C) Pairwise JSD distances between Subject A gut samples (A), Subject B gut samples (B), and Subject A saliva samples (C). (D-K) Median pairwise JSD as a function of sample temporal distance (blue points). The median value for each curve is shown as a solid red line. Asymptotic curves, which appear to converge on the solid red lines, are consistent with the notion of a stable microbiota over a given date range (D-F, H, K). Notably, pairwise JSD curves spanning distinct stable periods do not exhibit asymptotic behavior (G), or have relatively high asymptotes (J). (PDF 1966 kb) Additional file 4: Highly abundant OTUs are also persistent. Curves show the fraction of total reads (blue) and the fraction of total OTUs (green) accounted for by OTUs present in at least a given fraction of samples. Curves made using (A) Subject A gut samples from days 0 to 69 and 136 to 364, (B) Subject B gut samples from days 0 to 144, and (C) all Subject A saliva samples. (PDF 185 kb) Additional file 5: Fractional abundance of Enterobacteriaceae over time in Subject B’s gut. Each colored point represents the abundance of Enterobacteriaceae on a given date. Subject B suffered from a diarrheal illness from days 151 to 159 of the study, during which he was culture-positive for Salmonella. The Enterobacteriaceae, the parent family of Salmonella, account for a median of 0.004 % of daily reads over the entire time series. During days 151 to 159, this family comprises a median of 10.1 % of each day’s reads and peaks at 29.3 % of reads on day 159. (PDF 104 kb) Additional file 6: Bacteroidetes to Firmicutes ratio over time in Subject A’s gut. Subject A’s prolonged travel abroad shown in gray (days 71 to 122). The median Bacteroidetes/Firmicutes ratio in Subject A’s gut was 0.37 for days <70, 0.71 for days 90 to 103, and 0.38 for days >122. (PDF 138 kb) Additional file 7: Gut microbiota shifts across travel. Plotted over time is the Jensen-Shannon Distance (JSD) between Subject A gut microbiota and the median gut microbial community when the subject lived in the United States. Subject A left the United States on day 70 and returned on day 122 (travel period shaded in gray); he suffered from diarrheal illnesses between days 80 and 85 and days 104 and 113 (red shading). The red dashed line denotes the median JSD between domestic gut microbiota samples and the median domestic gut microbiota. The JSD increase after arriving abroad, but before the first diarrheal illness (days 71 to 79) argues that travel abroad was sufficient to alter Subject A’s gut microbiota. The JSD declines below the red median JSD line on day 136, suggesting that recovery of gut microbiota from travel required 14 days. (PDF 86 kb) Additional file 8: Statistics of host metadata dynamics. We measured day-to-day variability of host factors using the 1-day autocorrelation, which quantifies the correlation between a variable and its value the following day. (A) Autocorrelation of metadata variables tracked in Subjects A and B. Variables are colored by metadata category. Variables whose autocorrelation is only defined for one subject are shown using single-axis scatter plots. Most tracked host factors behaved randomly over time: the median autocorrelation across host factors was 0.14 in Subject A and 0.06 in Subject B. Exceptions to this trend were subject location, weight and body fat, which had autocorrelations >0.4 in both subjects. (B) Scatter plots of day-to-day variation among host factors with varying autocorrelation. Each point represents metadata value on a given day (t: x-axis) and the following day (t + 1: y-axis). (PDF 2186 kb) Additional file 9: All significant correlations (q <0.05) between subject metadata and microbiota. Table includes ‘redundant’ correlations involving similar food items and the same OTU cluster (for example, Food:Yogurt and Food:Yogurt:Non-Activia are both correlated with OTU cluster 84). A non-redundant list of correlations is presented in Table 1. (XLS 33 kb) Additional file 10: Taxonomy of Subject A saliva OTUs correlated with host metadata. Shown are oral bacterial taxa assigned to bacterial clusters that have significant correlations with host health or lifestyle (Table 1). Clusters were defined using hierarchical clustering (Additional file 16) of bacteria with similar temporal dynamics. Taxonomic IDs and Latin names are drawn from the Greengenes database. (XLS 14 kb) Additional file 11: Taxonomy of Subject A gut OTUs correlated with host metadata. Shown are gut bacterial taxa assigned to bacterial clusters that have significant correlations with host health or lifestyle (Table 1). Clusters were defined using hierarchical clustering (Additional file 16) of bacteria with similar temporal dynamics. Taxonomic IDs and Latin names are drawn from the Greengenes database. (XLS 47 kb) Additional file 12: Description of host metadata categories. (PDF 46 kb) Additional file 13: Flowchart of time series analysis pipeline for detecting OTU-metadata correlations. We implemented normalization, low-abundance OTU filtering, and autocorrelation elimination steps to reduce the likelihood of inferring spurious correlations. We also used OTU filtering and clustering to reduce the number of hypothetical OTU-metadata interactions tested. (PDF 65 kb) Additional file 14: Regression-based normalization example. (A) A toy community with five OTUs (blue dots) sampled on 2 different days is used to illustrate our normalization scheme. The Day 2 sample is sequenced twice as deeply as the Day 1 sample. Moreover, the community is unchanged across these days, except for one OTU that decreases by 90 % on Day 2 (arrowed). Our normalization technique uses regression to infer relative differences in total bacterial abundance between samples. We use median-based line-fitting (blue line), which is robust to outliers and whose slope reflects the two-fold difference in sequencing depth. A standard least-squares regression through the origin is affected by the OTU with sharply decreased abundance. (B) Day 2 OTUs rescaled by the robust regression scaling factor are unchanged relative to Day 1 (blue dots). By contrast, rescaling Day 2’s OTUs with standard techniques (Day 2 OTU levels sum to Day 1 OTU levels; green dots) causes artifactual day-to-day changes among four OTUs (arrowed). (PDF 124 kb) Additional file 15: Results of testing normalization with four simulated datasets. (A) We simulated bacterial communities over time using Ornstein-Uhlenbeck processes fit to observed gut bacterial dynamics in Subject B. Total bacterial load among the synthetic communities varied over time (blue line; mean-centered on 1). We simulated 16S sequencing runs using the synthetic time series, and input the runs into our robust regression-based normalization. Bacterial load estimated from the normalized communities (red line) closely tracked the simulated bacterial load, suggesting our normalization scheme is accurate. (B) Even after accounting for autocorrelations in bacterial load over time (see Methods section on Autocorrelation elimination), we observed significant correlations between the simulated and inferred bacterial loads. (PDF 333 kb) Additional file 16: Hierarchical clustering of detrended OTUs. Matrices show pairwise Spearman correlations between detrended OTU time series. Red points correspond with OTU pairs sharing more positive correlations, and blue points correspond with OTU pairs sharing more negative pairwise correlations. We used correlation matrices to perform hierarchical clustering. Clustered OTUs are segregated on this hierarchy by line color. Clustering yielded 138, 90, and 46 OTU clusters for Subject A’s gut, Subject B’s gut, and Subject A’s saliva time series, respectively. These clusters were ultimately tested against host factors to detect microbiota-lifestyle interactions (Table 1). (PDF 1027 kb) Additional file 17: Hierarchical clustering of OTUs across disturbances. Matrices show pairwise Spearman correlations between OTUs tracked before and after Subject A’s prolonged travel abroad or Subject B’s acute enteric infection. Red points correspond with OTU pairs sharing more positive correlations, and blue points correspond with OTU pairs sharing more negative pairwise correlations. We used correlation matrices to perform hierarchical clustering. Clustered OTUs are segregated on this hierarchy by line color. Clustering yielded 11 OTU clusters for both time series. Clusters here were used to analyze subjects’ gut microbiota response to large perturbations (Figures 3 and 4). (PDF 320 kb) Additional file 18: Sample and nutritional metadata. Metadata are provided for nucleotide sequences deposited on the EBI/ENA database under accession number ERP006059. These metadata include Subject A’s nutritional data, provided for the day preceding each sample. (CSV 914 kb)
