# Supplementary material for: Host lifestyle affects human microbiota on daily timescales
Source: Genome Biol. 2014 Jul 25;15(7):R89. doi: 10.1186/gb-2014-15-7-r89 (PMC4405912; doi:10.1186/gb-2014-15-7-r89)
Supplement: Supplementary file 3 — Additional file 3: Microbiota similarity over time measured with the Jensen-Shannon Distance (JSD). (A-C) Pairwise JSD distances between Subject A gut samples (A), Subject B gut samples (B), and Subject A saliva samples (C). (D-K) Median pairwise JSD as a function of sample temporal distance (blue points). The median value for each curve is shown as a solid red line. Asymptotic curves, which appear to converge on the solid red lines, are consistent with the notion of a stable microbiota over a given date range (D-F, H, K). Notably, pairwise JSD curves spanning distinct stable periods do not exhibit asymptotic behavior (G), or have relatively high asymptotes (J). (PDF 47 KB) [file 13059_2013_3286_MOESM3_ESM.pdf]

Descriptions of host metadata categories:

<u>Ailments</u>

Subjects could choose the following ailments from a drop-down list: chest pain, chills, cold sweat, cough (dry), cough (wet), dizziness, fatigue, fever, headache, hemorrhoids, muscle ache, nausea, post nasal drip, runny nose, shortness of breath, sore throat, stomach gas, stomach pains, stuffy nose, and vomiting. Subjects were also free to write in unlisted ailments.

<u>Bowel movements</u>

Subjects recorded each bowel movement and graded its density and weight according to the King's Stool Chart {Whelan:2008jt}. Subjects also recorded stool color and scored its odor (1: mild, 2: strong, 3:severe).

<u>Daily notes</u>

Subjects were provided with an empty form to record noteworthy daily events that did not fall into one of the other metadata categories.

<u>Diet</u>

Subjects kept simple food diaries each day. We automatically parsed the diaries into a hierarchy of 272 food categories using a rule-based text-mining routine inspired by a standard food frequency questionnaire {Rimm:1992tc} and implemented using Python and the OmniOutliner software (The Omni Group, Seattle, Washington). This questionnaire grouped foods into 7 broad categories: "vegetables," "fruits," "eggs, meta, etc.," "sweets, baked goods, misc.," "dairy," "breads, cereals, and staches," and "beverages." From days 1 to 72, and from days 123 to 202 of the study, Subject A also manually tracked his nutritional intake using the CalorieKing Nutrition Manager

(Wellness Solutions, Inc), which enabled quantitative measurement of daily micro and macronutrient intake.

Exercise

Subjects recorded when and how they exercised. Exercise duration was recorded in minutes and intensity was graded from 1 (low) to 5 (high).

Fitness

Subjects were asked to measure their weight and cardiovascular health each morning and evening. We provided subjects with digital body fat monitors and scales (Omron HBF-400) and digital blood pressure monitors (Omron HEM-780). Subjects used these devices to measure weight, body fat, heart rate, and systolic and diastolic blood pressure.

Location change

Subjects used this form to report whenever they left from and returned to their home state, or traveled internationally.

Medication

Subjects used this form to report whenever they consumed medication or prescription drugs.

Mood

Subjects were asked to rate their energy, stress, and happiness levels on a scale from 1 (low) to 5 (high) each morning and evening.

Oral hygiene

Subjects recorded when they used mouthwash, brushed their teeth, flossed, or rinsed with PBS for saliva sampling.

Sleep

Subjects recorded what time they slept and woke up. Subjects were instructed to record all sleep events, including naps. Subjects rated their sleep according to a 4-point scale (1: poor, 2: average, 3: good, 4: excellent). We parsed sleep logs to note if overnight sleep was interrupted or began prior to midnight.

Urination

Subjects recorded when they urinated. Subjects rated urine color using a 4-point scale (1: clear, 2: pale yellow, 3: yellow, 4: dark yellow) and duration with a 3-point scale (1: <20 seconds, 2: 20-40 seconds, 3: >40 seconds).

Vitamin intake

Subjects recorded whether or not they consumed a multivitamin.
