# Supplementary material for: Host lifestyle affects human microbiota on daily timescales
Source: Genome Biol. 2014 Jul 25;15(7):R89. doi: 10.1186/gb-2014-15-7-r89 (PMC4405912; doi:10.1186/gb-2014-15-7-r89)
Supplement: Supplementary file 4 — Additional file 4: Highly abundant OTUs are also persistent. Curves show the fraction of total reads (blue) and the fraction of total OTUs (green) accounted for by OTUs present in at least a given fraction of samples. Curves made using (A) Subject A gut samples from days 0 to 69 and 136 to 364, (B) Subject B gut samples from days 0 to 144, and (C) all Subject A saliva samples. (PDF 66 KB) [file 13059_2013_3286_MOESM4_ESM.pdf]

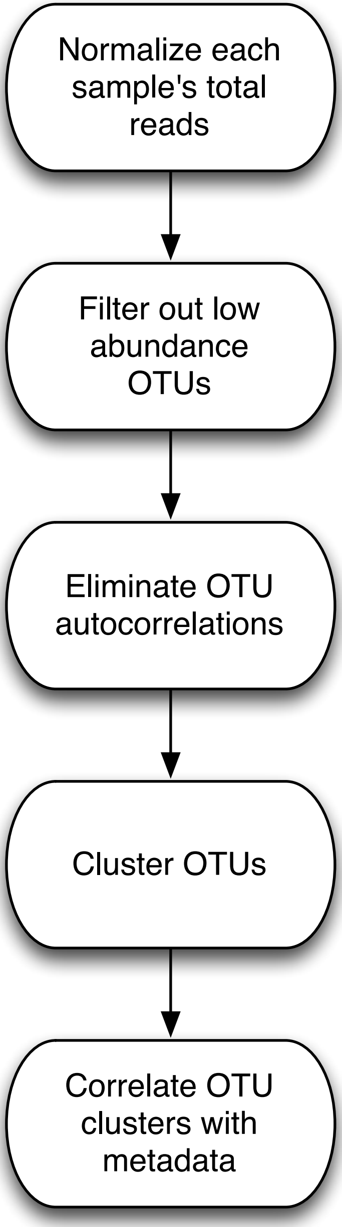
Normalize each sample's total reads
Filter out low abundance OTUs
Eliminate OTU autocorrelations
Cluster OTUs
Correlate OTU clusters with metadata
