# Supplementary figures and images for: Host lifestyle affects human microbiota on daily timescales
Source: Genome Biol. 2014 Jul 25;15(7):R89. doi: 10.1186/gb-2014-15-7-r89 (PMC4405912; doi:10.1186/gb-2014-15-7-r89)

**A**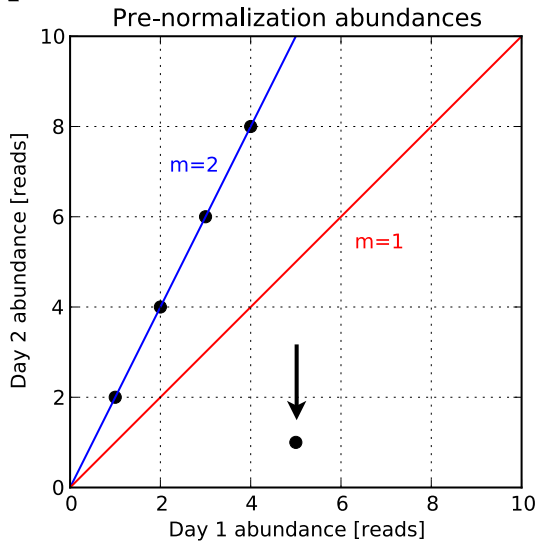**B**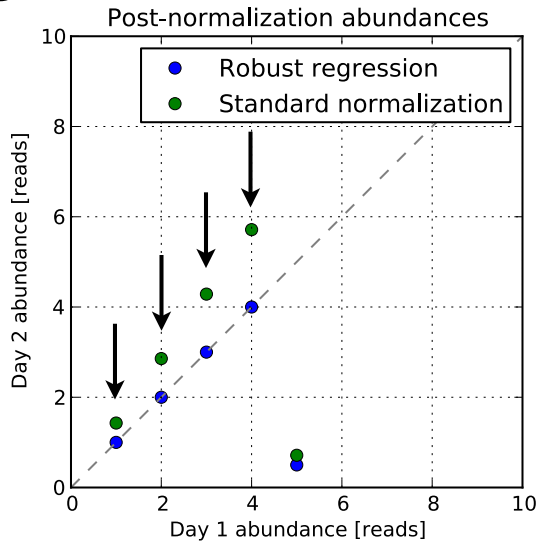

Supplement: Supplementary file 5 — Additional file 5: Fractional abundance of Enterobacteriaceae over time in Subject B’s gut. Each colored point represents the abundance of Enterobacteriaceae on a given date. Subject B suffered from a diarrheal illness from days 151 to 159 of the study, during which he was culture-positive for Salmonella. The Enterobacteriaceae, the parent family of Salmonella, account for a median of 0.004% of daily reads over the entire time series. During days 151 to 159, this family comprises a median of 10.1% of each day’s reads and peaks at 29.3% of reads on day 159. (PDF 125 KB) [file 13059_2013_3286_MOESM5_ESM.pdf]

**A**

Simulation 1

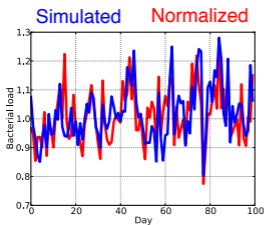

Simulation 2

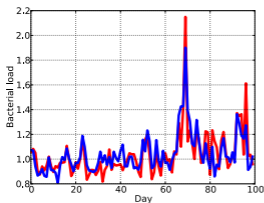

Simulation 3

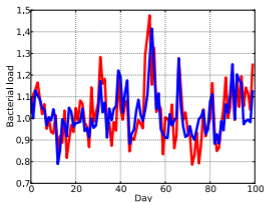

Simulation 4

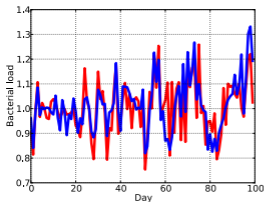**B**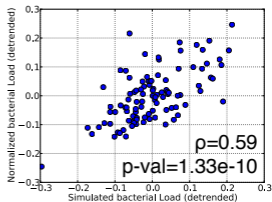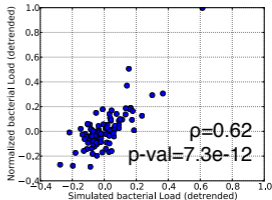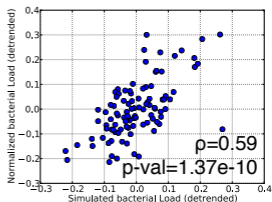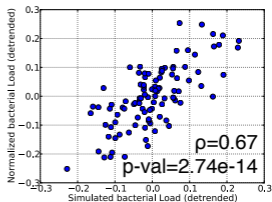

Supplement: Supplementary file 6 — Additional file 6: Bacteroidetes to Firmicutes ratio over time in Subject A’s gut. Subject A’s prolonged travel abroad shown in gray (days 71 to 122). The median Bacteroidetes/Firmicutes ratio in Subject A’s gut was 0.37 for days <70, 0.71 for days 90 to 103, and 0.38 for days >122. (PDF 333 KB) [file 13059_2013_3286_MOESM6_ESM.pdf]

Subject A  
Gut

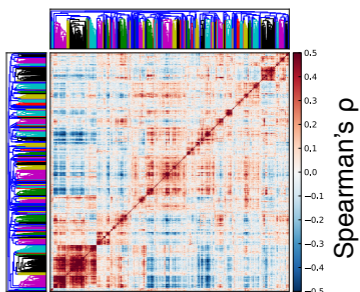

Subject B  
Gut

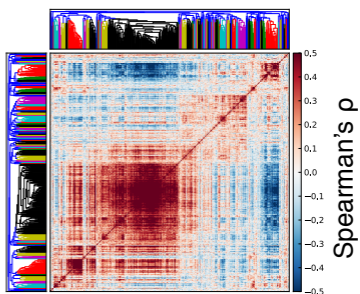

Subject A  
Saliva

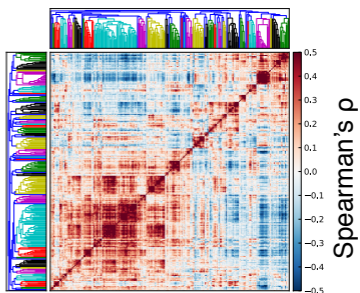

Supplement: Supplementary file 7 — Additional file 7: Gut microbiota shifts across travel. Plotted over time is the Jensen-Shannon Distance (JSD) between Subject A gut microbiota and the median gut microbial community when the subject lived in the United States. Subject A left the United States on day 70 and returned on day 122 (travel period shaded in gray); he suffered from diarrheal illnesses between days 80 and 85 and days 104 and 113 (red shading). The red dashed line denotes the median JSD between domestic gut microbiota samples and the median domestic gut microbiota. The JSD increase after arriving abroad, but before the first diarrheal illness (days 71 to 79) argues that travel abroad was sufficient to alter Subject A’s gut microbiota. The JSD declines below the red median JSD line on day 136, suggesting that recovery of gut microbiota from travel required 14 days. (PDF 1 MB) [file 13059_2013_3286_MOESM7_ESM.pdf]

# Subject A Gut (Days 40-153)

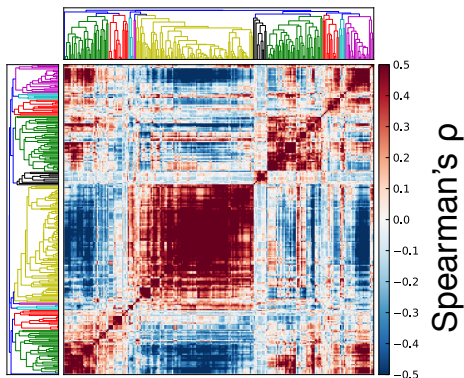

# Subject B Gut (Days 121-197)

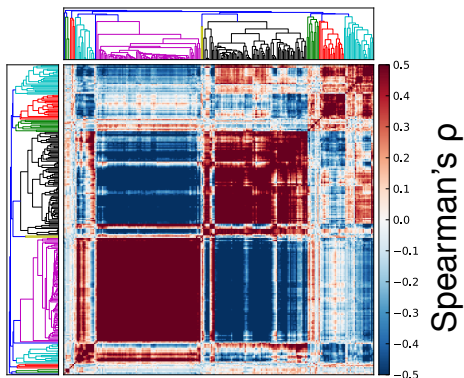

Supplement: Supplementary file 8 — Additional file 8: Statistics of host metadata dynamics. We measured day-to-day variability of host factors using the 1-day autocorrelation, which quantifies the correlation between a variable and its value the following day. (A) Autocorrelation of metadata variables tracked in Subjects A and B. Variables are colored by metadata category. Variables whose autocorrelation is only defined for one subject are shown using single-axis scatter plots. Most tracked host factors behaved randomly over time: the median autocorrelation across host factors was 0.14 in Subject A and 0.06 in Subject B. Exceptions to this trend were subject location, weight and body fat, which had autocorrelations >0.4 in both subjects. (B) Scatter plots of day-to-day variation among host factors with varying autocorrelation. Each point represents metadata value on a given day (t: x-axis) and the following day (t + 1: y-axis). (PDF 320 KB) [file 13059_2013_3286_MOESM8_ESM.pdf]
