# Supplementary figures and images for: Erratum to: Host lifestyle affects human microbiota on daily timescales
Source: Genome Biol. 2016 May 31;17:117. doi: 10.1186/s13059-016-0988-y (PMC4888531; doi:10.1186/s13059-016-0988-y)

Subject A Saliva 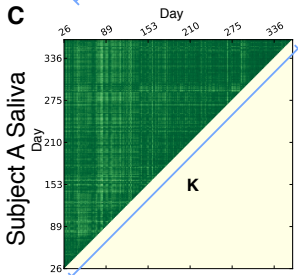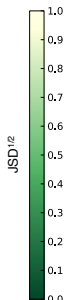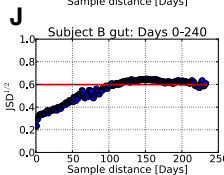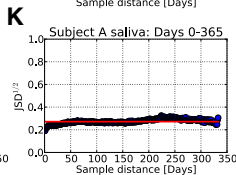

Supplement: Additional file 3: — Microbiota similarity over time measured with the Jensen-Shannon Distance (JSD). (A-C) Pairwise JSD distances between Subject A gut samples (A), Subject B gut samples (B), and Subject A saliva samples (C). (D-K) Median pairwise JSD as a function of sample temporal distance (blue points). The median value for each curve is shown as a solid red line. Asymptotic curves, which appear to converge on the solid red lines, are consistent with the notion of a stable microbiota over a given date range (D-F, H, K). Notably, pairwise JSD curves spanning distinct stable periods do not exhibit asymptotic behavior (G), or have relatively high asymptotes (J). (PDF 1966 kb) [file 13059_2016_988_MOESM3_ESM.pdf]

# A Donor A Gut

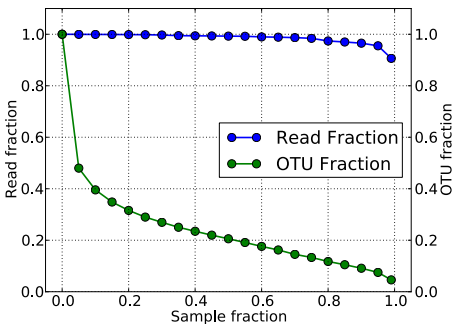

# B Donor B Gut

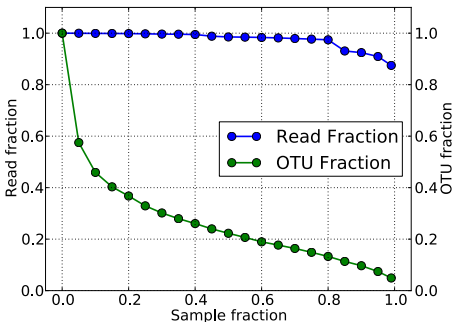

# C Donor A Saliva

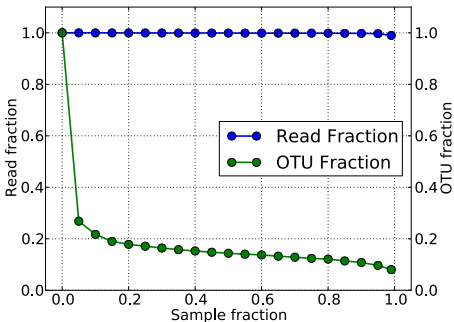

Supplement: Additional file 4: — Highly abundant OTUs are also persistent. Curves show the fraction of total reads (blue) and the fraction of total OTUs (green) accounted for by OTUs present in at least a given fraction of samples. Curves made using (A) Subject A gut samples from days 0 to 69 and 136 to 364, (B) Subject B gut samples from days 0 to 144, and (C) all Subject A saliva samples. (PDF 185 kb) [file 13059_2016_988_MOESM4_ESM.pdf]

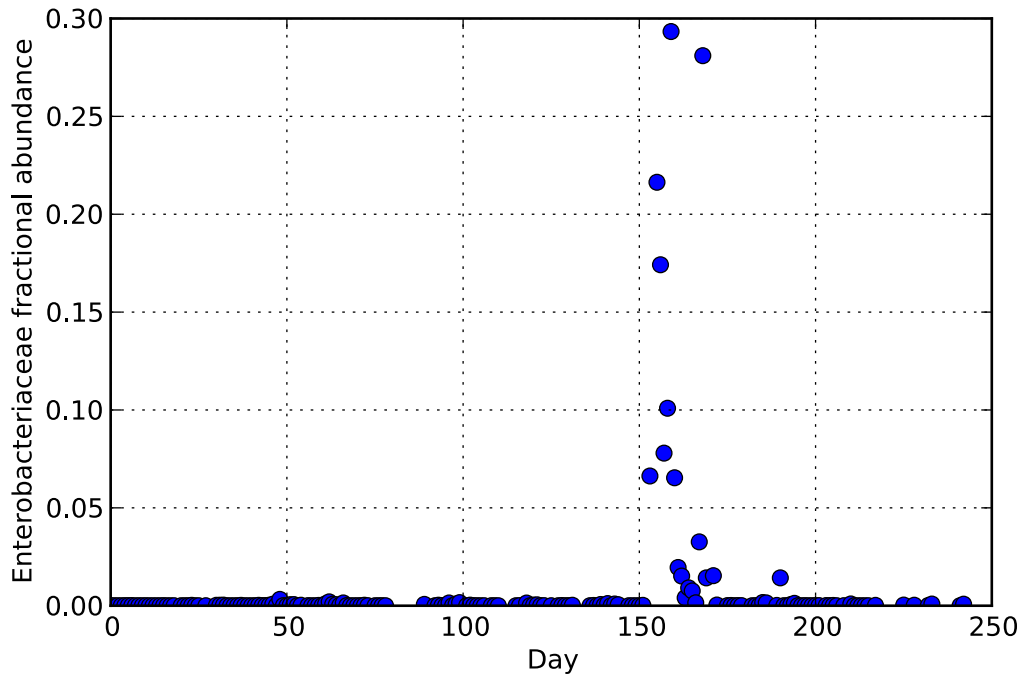

Supplement: Additional file 5: — Fractional abundance of Enterobacteriaceae over time in Subject B’s gut. Each colored point represents the abundance of Enterobacteriaceae on a given date. Subject B suffered from a diarrheal illness from days 151 to 159 of the study, during which he was culture-positive for Salmonella. The Enterobacteriaceae, the parent family of Salmonella, account for a median of 0.004 % of daily reads over the entire time series. During days 151 to 159, this family comprises a median of 10.1 % of each day’s reads and peaks at 29.3 % of reads on day 159. (PDF 104 kb) [file 13059_2016_988_MOESM5_ESM.pdf]

Subject A gut Bacteroidetes/Firmicutes ratio over time

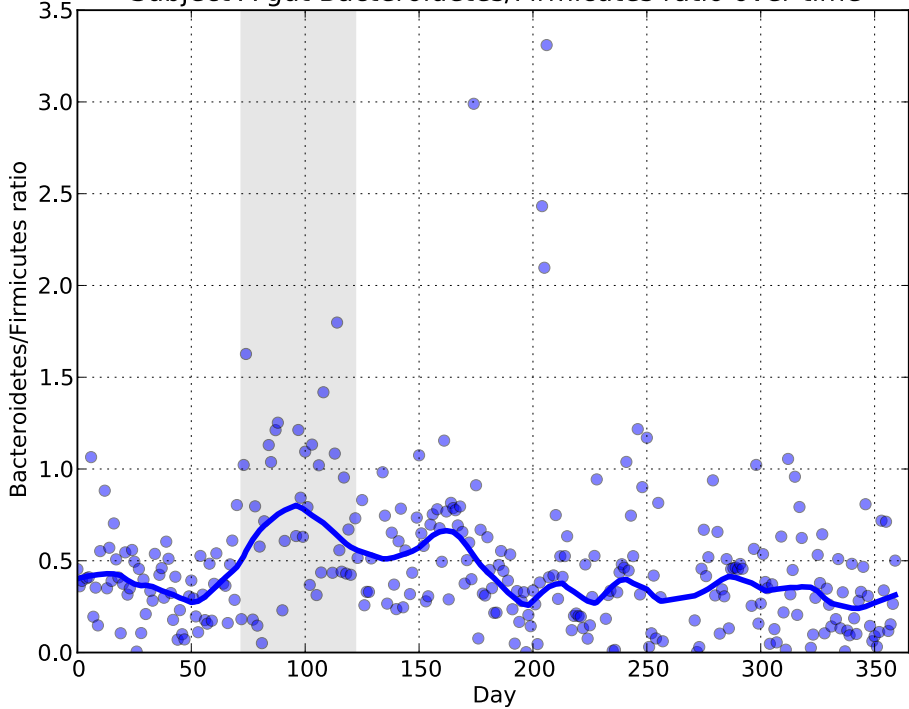

Supplement: Additional file 6: — Bacteroidetes to Firmicutes ratio over time in Subject A’s gut. Subject A’s prolonged travel abroad shown in gray (days 71 to 122). The median Bacteroidetes/Firmicutes ratio in Subject A’s gut was 0.37 for days <70, 0.71 for days 90 to 103, and 0.38 for days >122. (PDF 138 kb) [file 13059_2016_988_MOESM6_ESM.pdf]

# Subject A similarity to US samples

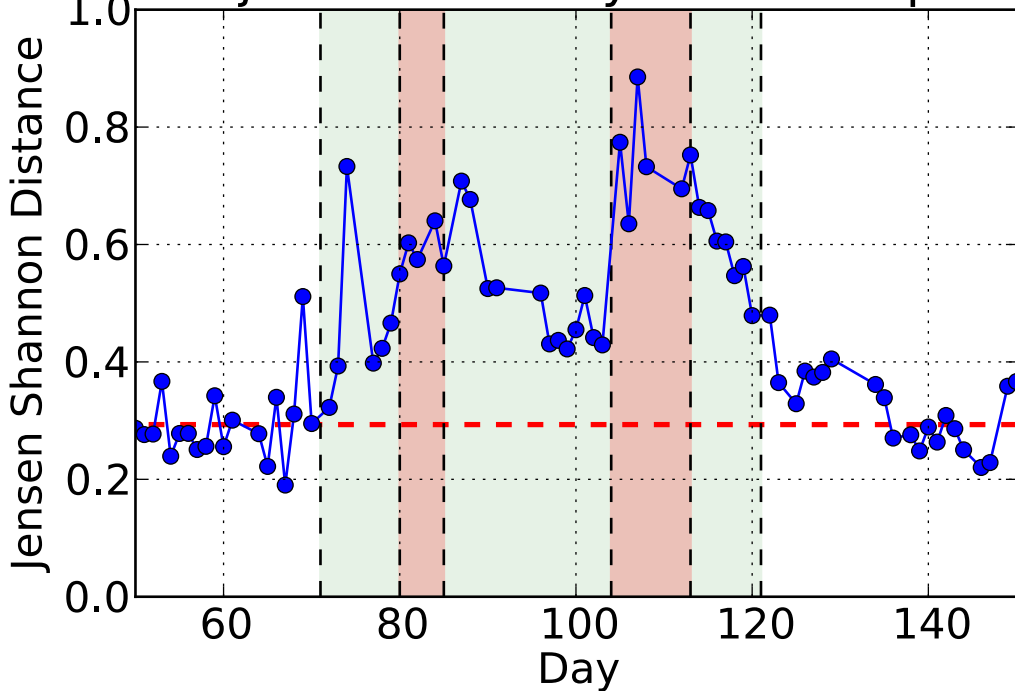

Supplement: Additional file 7: — Gut microbiota shifts across travel. Plotted over time is the Jensen-Shannon Distance (JSD) between Subject A gut microbiota and the median gut microbial community when the subject lived in the United States. Subject A left the United States on day 70 and returned on day 122 (travel period shaded in gray); he suffered from diarrheal illnesses between days 80 and 85 and days 104 and 113 (red shading). The red dashed line denotes the median JSD between domestic gut microbiota samples and the median domestic gut microbiota. The JSD increase after arriving abroad, but before the first diarrheal illness (days 71 to 79) argues that travel abroad was sufficient to alter Subject A’s gut microbiota. The JSD declines below the red median JSD line on day 136, suggesting that recovery of gut microbiota from travel required 14 days. (PDF 86 kb) [file 13059_2016_988_MOESM7_ESM.pdf]

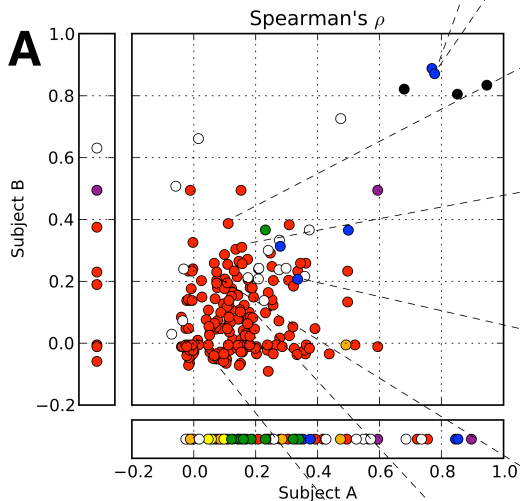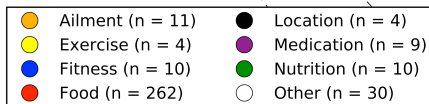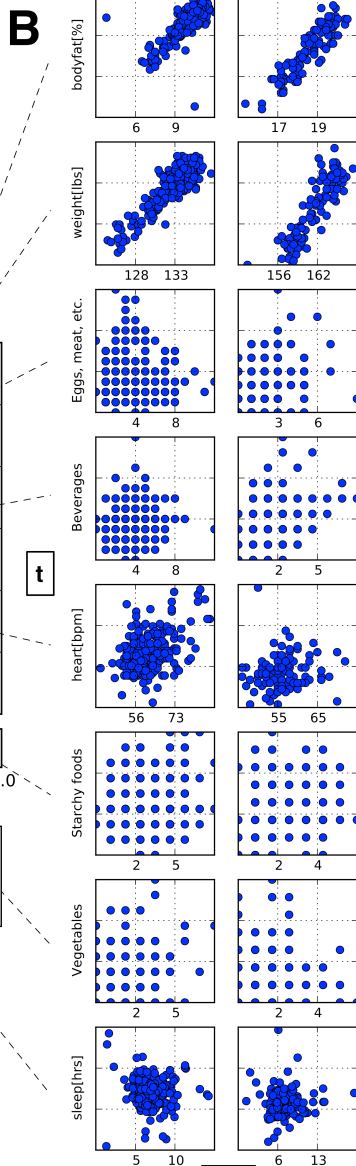

**t+1**

Supplement: Additional file 8: — Statistics of host metadata dynamics. We measured day-to-day variability of host factors using the 1-day autocorrelation, which quantifies the correlation between a variable and its value the following day. (A) Autocorrelation of metadata variables tracked in Subjects A and B. Variables are colored by metadata category. Variables whose autocorrelation is only defined for one subject are shown using single-axis scatter plots. Most tracked host factors behaved randomly over time: the median autocorrelation across host factors was 0.14 in Subject A and 0.06 in Subject B. Exceptions to this trend were subject location, weight and body fat, which had autocorrelations >0.4 in both subjects. (B) Scatter plots of day-to-day variation among host factors with varying autocorrelation. Each point represents metadata value on a given day (t: x-axis) and the following day (t + 1: y-axis). (PDF 2186 kb) [file 13059_2016_988_MOESM8_ESM.pdf]

**A**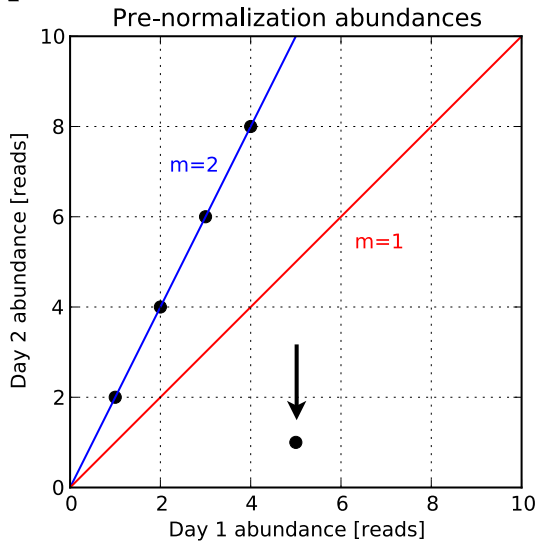**B**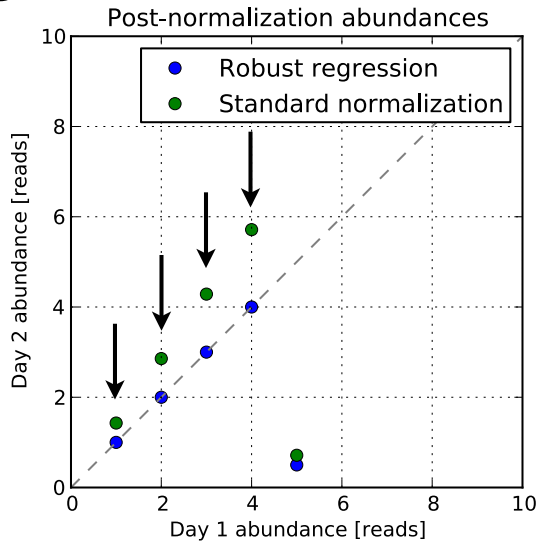

Supplement: Additional file 14: — Regression-based normalization example. (A) A toy community with five OTUs (blue dots) sampled on 2 different days is used to illustrate our normalization scheme. The Day 2 sample is sequenced twice as deeply as the Day 1 sample. Moreover, the community is unchanged across these days, except for one OTU that decreases by 90 % on Day 2 (arrowed). Our normalization technique uses regression to infer relative differences in total bacterial abundance between samples. We use median-based line-fitting (blue line), which is robust to outliers and whose slope reflects the two-fold difference in sequencing depth. A standard least-squares regression through the origin is affected by the OTU with sharply decreased abundance. (B) Day 2 OTUs rescaled by the robust regression scaling factor are unchanged relative to Day 1 (blue dots). By contrast, rescaling Day 2’s OTUs with standard techniques (Day 2 OTU levels sum to Day 1 OTU levels; green dots) causes artifactual day-to-day changes among four OTUs (arrowed). (PDF 124 kb) [file 13059_2016_988_MOESM14_ESM.pdf]

**A**

Simulation 1

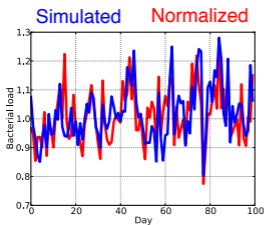

Simulation 2

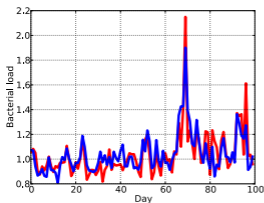

Simulation 3

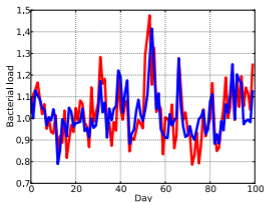

Simulation 4

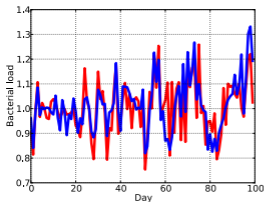**B**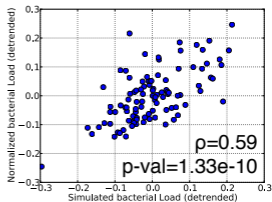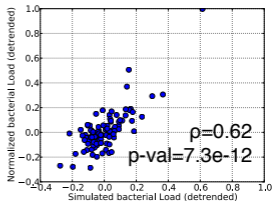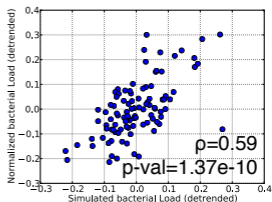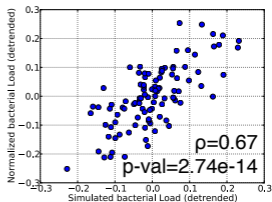

Supplement: Additional file 15: — Results of testing normalization with four simulated datasets. (A) We simulated bacterial communities over time using Ornstein-Uhlenbeck processes fit to observed gut bacterial dynamics in Subject B. Total bacterial load among the synthetic communities varied over time (blue line; mean-centered on 1). We simulated 16S sequencing runs using the synthetic time series, and input the runs into our robust regression-based normalization. Bacterial load estimated from the normalized communities (red line) closely tracked the simulated bacterial load, suggesting our normalization scheme is accurate. (B) Even after accounting for autocorrelations in bacterial load over time (see Methods section on Autocorrelation elimination), we observed significant correlations between the simulated and inferred bacterial loads. (PDF 333 kb) [file 13059_2016_988_MOESM15_ESM.pdf]

Subject A  
Gut

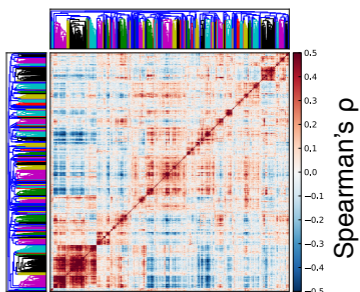

Subject B  
Gut

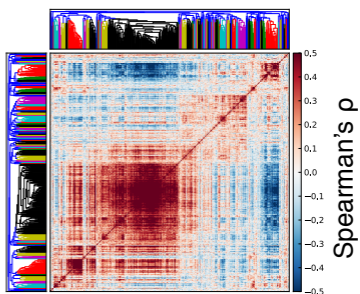

Subject A  
Saliva

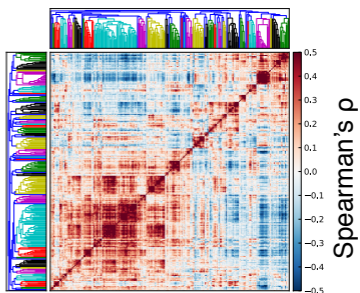

Supplement: Additional file 16: — Hierarchical clustering of detrended OTUs. Matrices show pairwise Spearman correlations between detrended OTU time series. Red points correspond with OTU pairs sharing more positive correlations, and blue points correspond with OTU pairs sharing more negative pairwise correlations. We used correlation matrices to perform hierarchical clustering. Clustered OTUs are segregated on this hierarchy by line color. Clustering yielded 138, 90, and 46 OTU clusters for Subject A’s gut, Subject B’s gut, and Subject A’s saliva time series, respectively. These clusters were ultimately tested against host factors to detect microbiota-lifestyle interactions (Table 1). (PDF 1027 kb) [file 13059_2016_988_MOESM16_ESM.pdf]

# Subject A Gut (Days 40-153)

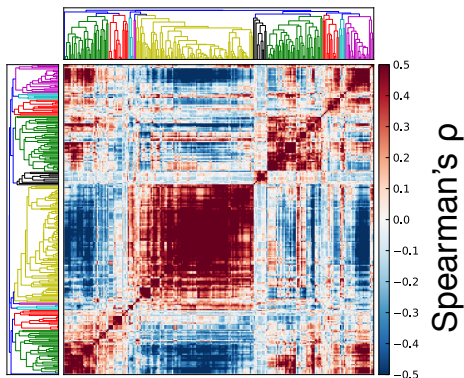

# Subject B Gut (Days 121-197)

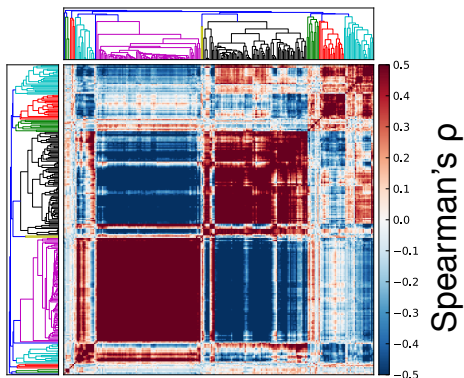

Supplement: Additional file 17: — Hierarchical clustering of OTUs across disturbances. Matrices show pairwise Spearman correlations between OTUs tracked before and after Subject A’s prolonged travel abroad or Subject B’s acute enteric infection. Red points correspond with OTU pairs sharing more positive correlations, and blue points correspond with OTU pairs sharing more negative pairwise correlations. We used correlation matrices to perform hierarchical clustering. Clustered OTUs are segregated on this hierarchy by line color. Clustering yielded 11 OTU clusters for both time series. Clusters here were used to analyze subjects’ gut microbiota response to large perturbations (Figures 3 and 4). (PDF 320 kb) [file 13059_2016_988_MOESM17_ESM.pdf]
